# Semiparametric regression based on quadratic inference function for multivariate failure time data with auxiliary information

**DOI:** 10.1007/s10985-020-09513-1

**Published:** 2021-01-08

**Authors:** Feifei Yan, Lin Zhu, Yanyan Liu, Jianwen Cai, Haibo Zhou

**Affiliations:** 1 School of Mathematics and Statistics, Huazhong University of Science and Technology, Wuhan, 430074 Hubei People’s Republic of China; 2 School of Mathematics and Statistics, Wuhan University, Wuhan, 430072 Hubei People’s Republic of China; 3 Department of Biostatistics, University of North Carolina at Chapel Hill, Chapel Hill, NC 27599-7420 USA; 4 School of Science, East China University of Technology, Nanchang, 330013 Jiangxi People’s Republic of China

**Keywords:** Multivariate failure time data, Validation sample, Quadratic inference function, Chi-squared test

## Abstract

This paper deals with statistical inference procedure of multivariate failure time data when the primary covariate can be measured only on a subset of the full cohort but the auxiliary information is available. To improve efficiency of statistical inference, we use quadratic inference function approach to incorporate the intra-cluster correlation and use kernel smoothing technique to further utilize the auxiliary information. The proposed method is shown to be more efficient than those ignoring the intra-cluster correlation and auxiliary information and is easy to implement. In addition, we develop a chi-squared test for hypothesis testing of hazard ratio parameters. We evaluate the finite-sample performance of the proposed procedure via extensive simulation studies. The proposed approach is illustrated by analysis of a real data set from the study of left ventricular dysfunction.

## Introduction

This paper is aimed at developing improved inference procedure for multivariate failure time data with auxiliary information. Large cohort studies often involve thousands or more subjects and the studies, especially when involving failure time outcomes, could last for many years. It is often that the measurement of the primary covariate can only be obtained for a random subset of the study cohort due to technical difficulties or financial limitations. On the other hand, some auxiliary information that is less precise but highly correlated to the primary exposure can be cheaply collected for all cohort members. The auxiliary information could be a mismeasured surrogate to the true covariate, or any covariate that is informative about the true covariate. An example is from the left ventricular dysfunction (SOLVD 1991) prevention study, which aims to assess the effects of risk factors on the time (possibly censored) to heart failure and the first myocardial infarction. One of the most important risk factors is patient’s ejection fraction (EF), which can be precisely measured by using a standardized radionucleotide technique, but the cost is very high. Therefore, EF is only measured on a randomly chosen subset of all cohort, while a less precise but cheaper measurement of EF was ascertained for all the patients using a nonstandardized technique. Because each patient could experience both heart failure and the first myocardial infarction, statistical methods for handling multivariate failure time data with covariate measurement error would be required.

Proper use of auxiliary information has been proved to lead to improved efficiency of survival estimates in multivariate failure time data with auxiliary information. For example, Hu and Lin (2004) proposed a corrected estimation function under the assumption that the error is symmetrically distributed. Liu et al. (2009) and Liu et al. (2010) developed estimated pseudo-partial likelihood method for multivariate failure time data with discrete and continuous auxiliary variable, respectively. Liu et al. (2012) and Fan and Wang (2009) studied this problem under the assumption that the intra-cluster subjects have common baseline hazard. The above studies are based on the marginal hazards model, the intra-cluster correlation, however, is ignored in the estimation procedures and only adjusted in the inference step by applying a robust sandwich variance estimate. The practice of ignoring the intra-cluster correlation would result in some loss of efficiency.

Some authors have proposed to incorporate correlation explicitly into the estimating equations to improve the efficiency of estimate in dealing with multivariate failure time data with covariates being fully observed. For example, Cai and Prentice (1995, 1997) added a weight matrix based on the inverse of correlation matrix of marginal martingales into the partial likelihood score equation. Simulation studies have shown that their approach is more efficient than that using independent structure when cluster size is small. However, their method is computation intensive when the cluster size is large because the computation involves an estimation of very high dimensional weighting correlation matrix. To overcome this shortcoming, Xue et al. (2010) developed a different approach by applying the method of quadratic inference function (QIF). Their method avoids to explicitly estimate the correlation parameters and is easy to implement especially when cluster size is large. We note that both these two methods assume that the covariates could be observed completely and therefore cannot be applied directly to SOLVD data.

Motivated by the advantages of the QIF method provided, we extend this method to the analysis of multivariate failure time data with auxiliary information. Here, we assume that the auxiliary covariate is continuous. We propose an estimated QIF method and study the asymptotic properties of the proposed estimator. The proposed method inherits the merit of QIF method which avoids the estimation of nuisance correlation parameters and is computationally easy to implement. Under certain regularity conditions, we establish the asymptotic normality of resulting estimator. Simulation studies show that our proposed method can improve the estimation efficiency compared with that ignoring dependent structure, such as the method by Liu et al. (2010). In addition, we study the problem of hypothesis testing, propose a proper test statistic which have a chi-squared limiting distribution under the null hypothesis.

The rest of the article is organized as follows. In Sect. 2, we introduce the model and describe the proposed estimation procedure. In Sect. 3, the large-sample properties of the proposed estimator are presented. In Sect. 4, a chi-squared test is developed for hypothesis testing. In Sect. 5, the finite-sample performance of the proposed procedures is assessed through extensive simulation studies. We illustrate the proposed method through analysis of a real data set from SOLVD study in Sect. 6. Some concluding remarks are given in Sect. 7 and the technical proofs are provided in “Appendix”.

## Model and estimation

### Preliminaries

Suppose that the whole cohort consists of *n* independent clusters, and each cluster contains *K* correlated failure types. Let (*i*, *k*) denote the *k*th $(k=1,\cdots , K)$ subject in the *i*th $(i=1,\cdots ,n)$ cluster. Let $\widetilde{T}_{ik}$ and $C_{ik}$ be potential failure time and censoring time for subject (*i*, *k*). With censoring, one observes $T_{ik} = \mathrm{min}(\widetilde{T}_{ik}, C_{ik})$ and $\Delta _{ik} = I(\widetilde{T}_{ik} \le C_{ik})$, where $I(\cdot )$ is the indicator function. Let $\widetilde{Z}_{ik}(t)$ be a *p*-vector of possibly time-dependent covariates.

For subject (*i*, *k*), the hazard function $\lambda _{ik}(t;\widetilde{Z}_{ik}(t))$ takes the following form:

2.1 $$\begin{aligned} \lambda _{ik}(t;\widetilde{Z}_{ik}(t))=\lambda _{0k}(t) \exp \{\beta ^{\mathrm{T}}\widetilde{Z}_{ik}(t)\}, \end{aligned}$$

where $\beta$ is a *p*-vector of unknown regression parameters and $\lambda _{0k}(t)$ is an unspecified marginal baseline hazard function pertaining to the *k*th failure type.

Note that model (2.1) includes as a special case the failure-type-specific model (Wei et al. 1989; Greene and Cai 2004) $\lambda _{ik}(t;\widetilde{Z}_{ik}(t)) =\lambda _{0k}(t)\exp \{\beta _k^{\mathrm{T}}\widetilde{Z}_{ik}(t)\}$, which allows for different covariate effect for different *k*. This can be seen by defining $\beta =(\beta _1^{\mathrm{T}},\ldots , \beta _k^{\mathrm{T}},\ldots ,\beta _K^{\mathrm{T}})^{\mathrm{T}}$ and $\widetilde{Z}_{ik}^{*}(t)=(\mathbf{0}^{\mathrm{T}}, \ldots , \widetilde{Z}_{ik}^{\mathrm{T}}(t),\ldots , \mathbf{0}^{\mathrm{T}} )^{\mathrm{T}}$ in the model $\lambda _{ik}(t;\widetilde{Z}_{ik}^{*}(t)) =\lambda _{0k}(t)\exp \{\beta ^{\mathrm{T}}\widetilde{Z}_{ik}^{*}(t)\}$. For simplicity, we write $\lambda _{ik}(t;\widetilde{Z}_{ik}(t))$ as $\lambda _{ik}(t)$ in the following.

Let $\Lambda _{0k}(t) = \int _{0}^{t} \lambda _{0k}(u) \mathrm{d}u$ be the marginal cumulative baseline hazard function for the *k*th failure type. Let $N_{ik}(t)= \Delta _{ik} I(T_{ik}\le t)$ and $Y_{ik}(t) =I(T_{ik} \ge t)$ be the observed counting process and the at-risk indicator process. For convenience, write the relative risk function as $r_{ik}(t; \beta ) = \exp \{\beta ^{\mathrm{T}}\widetilde{Z}_{ik} (t)\}$. Let $M_{ik}(t)=N_{ik}(t)-\int _{0}^{t}Y_{ik}(u) r_{ik}(u; \beta _0)\lambda _{0k}(u)\mathrm{d}u$ be the marginal martingale process, where $\beta _0$ is the true parameter. Given $\beta$, $\Lambda _{0k}(t)$ can be estimated consistently by the following Breslow type estimator (Breslow 1972):

2.2 $$\begin{aligned} \widetilde{\Lambda }_{0k}(t;\beta ) = \int _{0}^{t} \frac{\sum _{i=1}^{n} \mathrm{d}N_{ik}(u)}{\sum _{i=1}^{n} Y_{ik}(u) r_{ik}( u;\beta )}. \end{aligned}$$

Given $$\beta _0$$, it follows that $$ M_{ik}(t)$$ could be estimated as follows:

$$\begin{aligned} \widetilde{M}_{ik}(t;\beta _0)=N_{ik}(t)-\int _{0}^{t}Y_{ik}(u) r_{ik}(u;\beta _0) \widetilde{\Lambda }_{0k}(\mathrm{d}t;\beta _0). \end{aligned}$$

Let $\tau$ be the end time of study. Write $\widetilde{\varvec{M}}_i(t;\beta ) =(\widetilde{M}_{i1} (t;\beta ), \ldots , \widetilde{M}_{iK}(t;\beta ))^{\mathrm{T}}$. To improve the estimation efficiency, Cai and Prentice (1995) added proper weight matrix to the pseudo-partial likelihood equation, and proposed to obtain estimate of $\beta$ through solving the following equation:

2.3 $$\begin{aligned} \sum _{i=1}^{n} \int _{0}^{\tau } \varvec{R}_i^{\mathrm{T}} (t;\beta ) W_i(t;\beta ) \widetilde{\varvec{M}}_i(\mathrm{d} t; \beta ) = 0, \end{aligned}$$

where $W_i(t;\beta )=\widehat{corr}^{-1} (\varvec{M}_i({\varvec{T}}_i; \beta ))$ is the weight matrix with

$$\begin{aligned} \varvec{M}_i({\varvec{T}}_i; \beta ) = (M_{i1}(T_{i1}; \beta ), \ldots , M_{iK}(T_{iK}; \beta ))^{\mathrm{T}}, \end{aligned}$$

and

$$\begin{aligned} \varvec{R}_i^{\mathrm{T}}(t;\beta )=\left( \frac{ r_{i1}^{(1)} (t;\beta )}{ r_{i1}(t;\beta )}, \ldots , \frac{ r_{iK}^{(1)} (t;\beta )}{ r_{iK}(t;\beta )}\right) , \end{aligned}$$

with $r^{(j)}(t;\beta )$ denotes the *j*th derivative of $r(t;\beta )$ with respect to $\beta$. The weight matrix measures the intra-cluster correlation and is important to improve estimation efficiency. However, when the cluster size *K* is large, the estimation of weight matrix is computationally expensive. To overcome this shortcoming, Xue et al. (2010) proposed a QIF method which is based on the following generalized estimating equation:

2.4 $$\begin{aligned} \sum _{i=1}^{n} \int _{0}^{\tau } \varvec{R}_i^{\mathrm{T}}(t;\beta ) \Xi _i^{1/2}(t; \beta ) {\varvec{\Sigma }}_i^{-1}(\alpha ) \Xi _i^{-1/2}(t; \beta ) \widetilde{\varvec{M}}_i(\mathrm{d} t; \beta )=0, \end{aligned}$$

where $\Xi _i(t; \beta )= \mathrm{diag}\{\lambda _{i1}(t), \ldots , \lambda _{iK}(t)\}$ and $\varvec{\Sigma }_i(\alpha )$ is the working correlation matrix whose common structure is specified by a vector of nuisance correlation parameters $\alpha$. The inverse of the working correlation is approximated by a linear combination of several pre-specified symmetric basis matrices, namely,

2.5 $$\begin{aligned} \Sigma ^{-1} \approx a_1B_1 + \ldots + a_LB_L, \end{aligned}$$

where $B_1, \dots , B_L$ are known basis matrices and $a_1, \dots , a_L$ are unknown coefficients.

Substituting (2.5) in (2.4) leads to a linear combination of the elements of the following vector

$$\begin{aligned} \varvec{G}_n(\beta )&= \frac{1}{n} \sum _{i=1}^{n} \varvec{g}_i(\beta )\\&=\frac{1}{n} \sum _{i=1}^{n} \left( \begin{array}{c} \int _{0}^{\tau } \varvec{R}_i^{\mathrm{T}}(t;\beta ) \Xi _i^{1/2}(t; \beta ) B_1 \Xi _i^{-1/2}(t; \beta ) \widetilde{\varvec{M}}_i(\mathrm{d} t; \beta )\\ \vdots \\ \int _{0}^{\tau } \varvec{R}_i^{\mathrm{T}}(t;\beta ) \Xi _i^{1/2}(t; \beta ) B_L \Xi _i^{-1/2}(t; \beta ) \widetilde{\varvec{M}}_i(\mathrm{d}t; \beta ) \end{array}\right) . \end{aligned}$$

As there are more equations than unknown parameters, Xue et al. (2010) proposed to estimate $\beta$ by minimizing the following QIF:

2.6 $$\begin{aligned} \varvec{Q}_n(\beta ) = \varvec{G}_n^{\mathrm{T}}(\beta ) \varvec{W}_n^{-1}(\beta ) \varvec{G}_n(\beta ), \end{aligned}$$

where $\varvec{W}_n(\beta ) = \frac{1}{n^2} \sum _{i=1}^{n} \varvec{g}_i(\beta ) \varvec{g}_i^{\mathrm{T}}(\beta )$. We denote the solution as $\widetilde{\beta }_Q$ in the following.

In the implementation of QIF, there being an additional issue that the diagonal matrix $\Xi _i(t; \beta )$ involves the unknown baseline hazard function $\lambda _{0k}(t; \beta )$. Xue et al. (2010) suggested a kernel smoothed estimator $\widetilde{\lambda }_{0k}(t; \beta )$ as follows,

2.7 $$\begin{aligned} \widetilde{\lambda }_{0k}(t; \beta ) = \frac{1}{\nu _k}\sum _{i=1}^{n} \kappa \left( \frac{t-T_{ik}}{\nu _k} \right) \Delta \widetilde{\Lambda }_{0k}(T_{ik}; \beta ), \end{aligned}$$

where $\kappa (\cdot )$ is the Epanechnikov kernel function with $\nu _k$ being the rule-of-thumb bandwidth, and $\Delta \widetilde{\Lambda }_{0k}(t; \beta )=\widetilde{\Lambda }_{0k}(t; \beta )-\widetilde{\Lambda }_{0k}(t-; \beta )$ with $\widetilde{\Lambda }_{0k}(t; \beta )$ being the Breslow estimator given in (2.2).

### Estimated QIF for marginal hazards model

Consider the situation that the primary covariate can only be ascertained in validation set. Let $\widetilde{Z}_{ik}(t)$ consist of two parts, $X_{ik}(t)$ and $Z_{ik}(t)$, where $X_{ik}(t)$ is the primary variable which can only be observed in the validation set and $Z_{ik}(t)$ is the vector of the remaining covariates that are measured precisely for the full cohort. Accordingly, write the true parameter as $\beta _0=(\beta _{10}, \beta _{20})$ with $\beta _{10}$ and $\beta _{20}$ pertaining to $X_{ik}(t)$ and $Z_{ik}(t)$, respectively. Denote *A*(*t*) as a time-dependent auxiliary variable for the primary covariate *X*(*t*). $A(\cdot )$ can be measured for all cohort members. Suppose that *A* provides no additional information to model given *X*, i.e.,

$$\begin{aligned} \lambda (t; X(t), Z(t), A(t)) \equiv \lambda (t; X(t), Z(t)). \end{aligned}$$

Use $\eta _{ik} = 1$ or 0 to indicate whether the subject (*i*, *k*) is in the validation set or not. Let $V_k = \{i : \eta _{ik} = 1\}$ and $\overline{V}_k = \{i : \eta _{ik} = 0\}$ denote the *k*th marginal validation set and non-validation set, respectively. Then the observed data are:

$$\begin{aligned} \begin{array}{rl} \{T_{ik}, \Delta _{ik}, \eta _{ik}, Y_{ik}(t), X_{ik}(t), Z_{ik}(t), A_{ik}(t)\} &{} \quad \text{ if } i\in V_k\\ \{T_{ik}, \Delta _{ik}, \eta _{ik}, Y_{ik}(t), Z_{ik}(t), A_{ik}(t)\} &{} \quad \text{ if } i \in \overline{V}_k. \end{array} \end{aligned}$$

According to Liu et al. (2009), when subject (*i*, *k*) is in non-validation set, the hazard function given observed data can be written as:

$$\begin{aligned} \lambda _{ik}(t; Z_{ik}(t), A_{ik}(t))&= \lambda _{0k}(t) e^{\beta _2^{\mathrm{T}} Z_{ik}(t)}E\{e^{\beta _1^{\mathrm{T}}X_{ik}(t)}| Y_{ik}(t)=1, A_{ik}(t), Z_{ik}(t)\}\\&= \lambda _{0k}(t) e^{\beta _2^{\mathrm{T}}Z_{ik}(t)}E\{e^{\beta _1^{\mathrm{T}}X_{ik}(t)}| Y_{ik}(t)=1, A_{ik}^{*}(t)\}, \end{aligned}$$

where $A_{ik}^{*}(t)$ denotes all the possible auxiliary information, which may include the auxiliary covariate $A_{ik}(t)$ and the part from $Z_{ik}(t)$. Therefore, the induced relative risk function is

$$\begin{aligned} r_{ik}(t;\beta ) = \varphi _{ik}( t;\beta )\eta _{ik} +\psi _{ik}(t;\beta )(1-\eta _{ik}), \end{aligned}$$

where $$\varphi _{ik}(t;\beta ) = \exp \{\beta _1^{\mathrm{T}} X_{ik}(t)+\beta _2^{\mathrm{T}}Z_{ik}(t)\}$$, and

$$\begin{aligned} \psi _{ik}(t;\beta ) = \exp \{\beta _2^{\mathrm{T}}Z_{ik}(t)\} E\{e^{\beta _1^{\mathrm{T}}X_{ik}(t)}| Y_{ik}(t)=1, A_{ik}^{*}(t)\}. \end{aligned}$$

If the conditional density of $X_{ik}$, written as $f( x|T_{ik}\ge t, A_{ik}^*)$, is a known function up to a parameter $\theta$, then $(\beta ,\theta )$ can be estimated by using the induced risk function to replace risk function in equations (2.3) or (2.4). However, misspecification of such parameterization may lead to biased estimates. We use empirical method to estimate $\psi _{ik}(t;\beta )$ and then replace it with the corresponding estimate.

In this paper, we consider the often encountered case that both the primary covariate $X_{ik}(t)$ and the auxiliary variable $A_{ik}^*(t)$ are one-dimensional. The unknown part of induced relative risk function in non-validation set is estimated by kernel smoothing method

2.8 $$\begin{aligned} \widehat{\psi }_{ik}(t; \beta ) = \frac{\sum _{j \in V_k}Y_{jk}(t) \Psi \{\mu _k^{-1}(A_{jk}^{*}(t)-A_{ik}^{*}(t))\} e^{\beta _1 X_{jk}(t)}}{\sum _{j \in V_k}Y_{jk}(t) \Psi \{\mu _k^{-1}(A_{jk}^{*}(t)-A_{ik}^{*}(t))\}} e^{\beta _2^{\mathrm{T}}Z_{ik}(t)}, \end{aligned}$$

where $\Psi (\cdot )$ is a kernel function, $\mu _k$ is the bandwidth. Imputation of the relative risk by interpolation would be used when the denominator is 0. Therefore, the estimate of the relative risk is

$$\begin{aligned} \widehat{r}_{ik}(t; \beta ) = \varphi _{ik}(t; \beta )\eta _{ik} +\widehat{\psi }_{ik}(t; \beta )(1-\eta _{ik}). \end{aligned}$$

Replacing $r_{ik}(t; \beta )$ by $\widehat{r}_{ik}(t; \beta )$ in the notations in Sect. 2.1, we obtain an estimated version of $\varvec{R}_i, \widetilde{\Lambda }_{0k}, \lambda _{0k}, \Xi _i, \varvec{\widetilde{M}}_i, \varvec{g}_i$ and $\varvec{G}_n$. To differentiate, write as $\widehat{\varvec{R}}_i, \widehat{\Lambda }_{0k}, \widehat{\lambda }_{0k}, \widehat{\Xi }_i$, $\varvec{\widehat{M}}_i, \widehat{\varvec{g}}_i$ and $\varvec{\widehat{\varvec{G}}}_n$. It yields an estimated QIF as

$$\begin{aligned} \widehat{\varvec{Q}}_n(\beta ) = \widehat{\varvec{G}}_n^{\mathrm{T}}(\beta ) \widehat{\varvec{W}}_n^{-1}(\beta ) \widehat{\varvec{G}}_n(\beta ), \end{aligned}$$

where $\widehat{\varvec{W}}_n(\beta ) = \frac{1}{n^2} \sum _{i=1}^{n} \widehat{\varvec{g}}_i(\beta ) \widehat{\varvec{g}}_i^{\mathrm{T}}(\beta )$. $\beta _0$ can be estimated by minimizing $\widehat{Q}_n(\beta )$, i.e.,

2.9 $$\begin{aligned} \widehat{\beta }_Q = \underset{\beta }{\mathrm{argmin}} \widehat{\varvec{Q}}_n(\beta ). \end{aligned}$$

To reduce the computation burden, we approximate the first and the second order derivatives of $\widehat{Q}_n(\beta )$ as in Qu et al. (2000) as follows.

$$\begin{aligned} \widehat{\varvec{Q}}_n^{(1)} (\beta )= & {} 2 \{\widehat{\varvec{G}}_n^{(1)}(\beta )\}^{\mathrm{T}} \widehat{\varvec{W}}_n^{-1} (\beta ) \widehat{\varvec{G}}_n(\beta )+o_p(1),\\ \widehat{\varvec{Q}}_n^{(2)} (\beta )= & {} 2\{\widehat{\varvec{G}}_n^{(1)}(\beta )\}^{\mathrm{T}} \widehat{\varvec{W}}_n^{-1} (\beta ) \widehat{\varvec{G}}_n^{(1)}(\beta )+o_p(1). \end{aligned}$$

Then, Newton-Raphson algorithm can be applied by using the approximation.

## Asymptotic properties

In this section, we present the asymptotic properties of the proposed estimated QIF estimator $\widehat{\beta }_Q$, and provide standard error formula for it.

Let $n_k$ denote the number of subjects in ${V}_k$ and assume $n_k/n \rightarrow \rho _k$ as $n \rightarrow \infty$, where $\rho _k$ represents the probability of subject (*i*, *k*) being sampled into the *k*th marginal validation set. Under the conditions listed in “Appendix”, we demonstrate the asymptotic behavior of $\widehat{\beta }_Q$ in the following theorems.

### Theorem 1

Under conditions (C1)–(C9) in “Appendix”, the following results hold:

(I) The proposed estimator $\widehat{\beta }_Q$ is a consistent estimator of $\beta _0$.
(II) $\sqrt{n}(\widehat{\beta }_Q - \beta _0)$ is asymptotically normally distributed with mean zero and variance matrix $\Sigma _Q(\beta _0)= (J_0^{\mathrm{T}} \varvec{W}_0^{-1}J_0)^{-1}$, where $J_0 = J(\beta _0)$, with
  $$\begin{aligned} J(\beta _0) = \left( \begin{array}{c} \Gamma (\beta _0, B_1)\\ \vdots \\ \Gamma (\beta _0, B_L) \end{array}\right) , \end{aligned}$$
  and $\Gamma (\beta _0, B)$ and $\varvec{W}_0$ are defined in (A.1) and (A.2) in “Appendix”, respectively.

The asymptotic covariance $\Sigma _Q(\beta _0)$ can be consistently estimated by

$$\begin{aligned} \widehat{\Sigma }_Q(\widehat{\beta }_Q) = \{ \widehat{J}^{\mathrm{T}} (\widehat{\beta }_Q) \widehat{\varvec{W}}^{-1} \widehat{J}(\widehat{\beta }_Q) \}^{-1}, \end{aligned}$$

where

$$\begin{aligned} \widehat{J}(\beta ) = \left( \begin{array}{c} \widehat{\Gamma }(\beta , B_1)\\ \vdots \\ \widehat{\Gamma }(\beta , B_L) \end{array}\right) , \end{aligned}$$

with

$$\begin{aligned} \widehat{\Gamma }(\beta , B)&= \frac{1}{n} \sum _{i=1}^{n} \sum _{k=1}^{K} \Delta _{ik} \left( \sum _{m=1}^{K} \frac{\partial \widehat{R}_{im}(T_{ik}; \beta ) \widehat{\phi }_{imk}(T_{ik};\beta , B)}{\partial \beta ^{\mathrm{T}}} -\frac{\widehat{S}_k^{3}(T_{ik}; \beta , B)}{\widehat{S}_k^{0}(T_{ik}; \beta )} \right. \\&\quad \left. -\frac{\widehat{S}_k^{4}(T_{ik}; \beta , B) +\widehat{S}_k^{5}(T_{ik}; \beta , B)}{\widehat{S}_k^{0}(T_{ik}; \beta )} +\frac{\widehat{S}_k^{2}(T_{ik}; \beta , B)(\widehat{S}_k^{1}(T_{ik}; \beta ))^{\mathrm{T}}}{(\widehat{S}_k^{0}(T_{ik}; \beta ))^2} \right) , \end{aligned}$$

and

$$\begin{aligned} \widehat{\varvec{W}} = \{\widehat{\varvec{\omega }} (\widehat{\beta }_Q, B_j, B_{j^{'}})\}_{j, j^{'} =1}^{L}, \end{aligned}$$

where

$$\begin{aligned} \widehat{\varvec{\omega }}(\beta , B_j, B_{j^{'}}) = n^{-1} \sum _{i=1}^{n} \sum _{k=1}^{K} \sum _{m=1}^{K} \widehat{U}_{ik} (\beta , B_j) \widehat{U}_{im}^{\mathrm{T}}(\beta , B_{j^{'}}), \end{aligned}$$

with

$$\begin{aligned} \widehat{U}_{ik}(\beta , B)&=\Delta _{ik}\widehat{\varvec{\zeta }}_{ik} (T_{ik};\beta , B)-\sum _{l=1}^{n} \Delta _{lk} \widehat{\varvec{\zeta }}_{ik}(T_{lk};\beta , B) \frac{Y_{ik}(T_{lk}) \widehat{r}_{ik}(T_{lk}; \beta )}{n\widehat{S}_k^{0}(T_{lk}; \beta )} \\&\quad - \frac{n-n_k}{n_k} \sum _{l=1}^{n} \Delta _{lk} \widehat{\varvec{\zeta }}_{ik}(T_{lk};\beta , B) \frac{Y_{ik}(T_{lk}) (\widehat{r}_{ik}(T_{lk}; \beta ) - \widehat{\psi }_{ik}(T_{lk}; \beta ))}{n \widehat{S}_k^{0}(T_{lk}; \beta )} , \end{aligned}$$

where $\widehat{S}_k^{d}\ (d=0,\ldots ,5)$ and $\widehat{\varvec{\zeta }}_{ik} (t;\beta , B)$ are defined in “Appendix”.

### Remark 1

As a special case of the estimated QIF estimator, the estimator using the independent working correlation is denoted as $\widehat{\beta }_I$, which is the same as the EPPL estimator of Liu et al. (2010), but different expressions of the variance matrix $\Sigma _I(\beta _0)$ of the asymptotic distribution of $\sqrt{n}(\widehat{\beta }_I - \beta _0)$ and its estimator $\widehat{\Sigma }_I(\widehat{\beta }_I)$ are provided under the conditions (C1)–(C8) in “Appendix”. From the corresponding expressions of $\Sigma _Q(\beta _0)$ and $\widehat{\Sigma }_Q(\widehat{\beta }_Q)$, we can obtain that

$$\begin{aligned} \Sigma _I(\beta _0) =\Gamma ^{-1}(\beta _0, I) \varvec{\omega }(\beta _0, I, I) \Gamma ^{-1}(\beta _0, I), \end{aligned}$$

and

$$\begin{aligned} \widehat{\Sigma }_I(\widehat{\beta }_I)=\widehat{\Gamma }^{-1}(\widehat{\beta }_I, I) \widehat{\varvec{\omega }} (\widehat{\beta }_I, I, I) \widehat{\Gamma }^{-1}(\widehat{\beta }_I, I). \end{aligned}$$

## Inference on hazard ratio parameters

The QIF is built on an objective function, which provides a natural way to make inference about the hazard ratio parameter $\beta$. Suppose that $\beta$ is partitioned into $\gamma$ and $\delta$, where $\gamma$ is vector of hazard ratio parameters of interest with dimension $p_0$, and $\delta$ is a vector of nuisance parameters with dimension $p-p_0$. As a special case, we also allow $p_0=p$, with $\beta = \gamma$ and $\delta$ being absent.

To test

$$\begin{aligned} H_0: \gamma =\gamma _0 \quad \text{ versus } \quad H_1: \gamma \ne \gamma _0, \end{aligned}$$

we propose a test statistic

4.1 $$\begin{aligned} T=\widehat{\varvec{Q}}_n(\gamma _0, \widetilde{\delta }) -\widehat{\varvec{Q}}_n(\widehat{\gamma }, \widehat{\delta } ), \end{aligned}$$

where

$$\begin{aligned} \widetilde{\delta } = \underset{\delta }{\mathrm{argmin}} \widehat{\varvec{Q}}_n(\gamma _0, \delta ), \quad (\widehat{\gamma }, \widehat{\delta }) = \underset{(\gamma , \delta )}{\mathrm{argmin}} \widehat{\varvec{Q}}_n(\gamma , \delta ). \end{aligned}$$

The values of $\widehat{\varvec{Q}}_n(\gamma _0, \widetilde{\delta })$ and $\widehat{\varvec{Q}}_n(\widehat{\gamma }, \widehat{\delta } )$ measure how well the model fits the data under $H_0$ and $H_1$, respectively. Under $H_0$, the difference between $\widehat{\varvec{Q}}_n(\gamma _0, \widetilde{\delta })$ and $\widehat{\varvec{Q}}_n(\widehat{\gamma }, \widehat{\delta })$ should be very small. However, under $H_1$, $\widehat{\varvec{Q}}_n(\gamma _0, \widetilde{\delta })$ should be systematically larger than $\widehat{\varvec{Q}}_n(\widehat{\gamma }, \widehat{\delta } )$.

### Theorem 2

Suppose conditions (C1)–(C9) in “Appendix” are satisfied, under $H_0$, the test statistic *T* asymptotically follows chi-squared distribution with $p_0$ degrees of freedom.

### Comment

To prove Theorem 2, we rewrite that

$$\begin{aligned} \widehat{\varvec{Q}}_n(\gamma _0, \widetilde{\delta }) -\widehat{\varvec{Q}}_n(\widehat{\gamma }, \widehat{\delta })&=(\widehat{\varvec{Q}}_n(\gamma _0, \widetilde{\delta }) - \varvec{Q}_n(\gamma _0, \widetilde{\delta }))-(\widehat{\varvec{Q}}_n(\widehat{\gamma }, \widehat{\delta }) -\varvec{Q}_n(\widehat{\gamma }, \widehat{\delta })) \\&\quad + (\varvec{Q}_n(\gamma _0, \widetilde{\delta }) -\varvec{Q}_n(\gamma _0, \widetilde{\delta }^*)) -(\varvec{Q}_n(\widehat{\gamma }, \widehat{\delta }) -\varvec{Q}_n(\widehat{\gamma }^*, \widehat{\delta }^*))\\&\quad +(\varvec{Q}_n(\gamma _0, \widetilde{\delta }^*) -\varvec{Q}_n(\widehat{\gamma }^*, \widehat{\delta }^*)), \end{aligned}$$

where $\varvec{Q}_n$ is defined as in (2.6), $\widetilde{\delta }^* = \underset{\delta }{\mathrm{argmin}} \varvec{Q}_n(\gamma _0, \delta )$, and $(\widehat{\gamma }^*, \widehat{\delta }^*) = \underset{(\gamma , \delta )}{\mathrm{argmin}} \varvec{Q}_n(\gamma , \delta )$. From the proof of Theorem 1, we can obtain that the first two brackets equal to $o_p(1)$. In addition, from the conclusions of the previous Theorem 1 and the Theorem 1 in Xue et al. (2010), both $\widehat{\beta }_Q$ and $\widetilde{\beta }_Q$ are consistent estimators of $\beta _0$, then we can have that the third and the fourth brackets also equal to $o_p(1)$. Furthermore, the last bracket asymptotically approaches to a random variable which follows chi-squared distribution with $p_0$ degrees of freedom, the proof of the last one is similar to the proof of Theorem 1 in Qu et al. (2000).

## Simulation studies

In this section, we conduct simulation studies to evaluate the finite-sample behavior of the proposed method. We first evaluate the performance of proposed estimator in Sect. 5.1 and then the performance of inference method in Sect. 5.2.

### Performance of estimated QIF estimator

We compare the proposed estimator with the QIF estimator proposed by Xue et al. (2010) based only on the validation set and the EPPL estimator ($\widehat{\beta }_I$) of Liu et al. (2010), which utilizes the auxiliary information but does not consider the intra-cluster correlation in the estimate of $\beta$. The proposed estimator takes both the intra-cluster correlation and the auxiliary information into account.

The covariates $(X_{i1}, X_{i2}, \ldots , X_{iK})$, which are only observed in the validation sets in the real studies, are generated independently from uniform distribution *U*(0, 1). The covariates $(Z_{i1}, Z_{i2}, \ldots , Z_{iK})$ are independent binary covariates taking value one with probability 0.5. The multivariate failure times $\widetilde{T}_{i1}, \widetilde{T}_{i2}, \ldots , \widetilde{T}_{iK}$ are generated from multivariate (Clayton and Cuzick 1985) model with the joint survival function

$$\begin{aligned} S(t_1, \ldots , t_K; D_1, \ldots , D_K) = \left( \sum _{k=1}^{K} \exp (\theta ^{-1}\lambda _{0k}t_ke^{\beta _k^{\mathrm{T}} D_k}) -(K-1)\right) ^{-\theta }, \end{aligned}$$

where $D_k=(X_k^{\mathrm{T}}, Z_k^{\mathrm{T}})^{\mathrm{T}}$, and $\beta _k$, which may vary with the failure type, is the corresponding parameter of $D_k$, and $\theta$ is the dependence parameter, a larger value of which represents a weaker dependence between the failure times. We set $\theta =0.25$, 0.5 or 2, which presents a varying degree of correlation between the generated failure times, and the baseline hazard function $\lambda _{0k}=1$. The simulated failure times $(t_1, \ldots , t_K)$ are generated by using the algorithm described in Cai and Shen (2000) through

$$\begin{aligned} t_1&= -e^{-\beta _1^{\mathrm{T}}D_1} \ln (1-u_1), \\ t_k&= \theta e^{-\beta _k^{\mathrm{T}} D_k} \ln \left[ (k-1)-\sum _{i=1}^{k-1}a_i+\left( \sum _{i=1}^{k-1}a_i-(k-2)\right) (1-u_k)^{-(\theta + k -1)^{-1}} \right] , \end{aligned}$$

for $k=2, \ldots , K$, where $a_i=\exp (\theta ^{-1}t_i e^{\beta _i^{\mathrm{T}} D_i})$ for $i=1, \dots , k-1$, and $(u_1,\ldots , u_K)$ are generated from uniform distribution over interval (0, 1). Censoring times are generated from *U*(0, *c*), where *c* is a selected constant to achieve a specified censoring rate.

Notice that the true correlation structure of the Clayton model is exchangeable, the working correlation in (2.9) is taken to be exchangeable, and the corresponding estimated QIF estimator is denoted as $\widehat{\beta }_{QE}$. We calculated another estimated QIF estimator $\widehat{\beta }_{QA}$ using the misspecified AR(1) working correlation. The corresponding resulting estimators of the QIF method based only on the validation set are denoted as $\widetilde{\beta }_{QEV}$ and $\widetilde{\beta }_{QAV}$, respectively.

To estimate the induced relative risk function and the baseline hazard function, we apply the Epanechnikov kernel function in (2.8) and (2.7) with bandwidths $\mu _k = 2 \widehat{\sigma }(A_{V_k})n_k^{-1/3}$ and $\nu _k = 1.06 \widehat{\sigma }(T_k)n^{-1/5}$, respectively, where $\widehat{\sigma }(\cdot )$ is the sample standard deviation function, $A_{V_k}$ is the part of auxiliary covariate $A_{k}$ in the *k*th marginal validation set. We choose the nearest neighbor interpolation to estimate the induced relative function when the denominator in (2.8) is 0. In addition, it is worth noting that $\widehat{\lambda }_{0k}(t)$ may be 0 at some locations because the Epanechnikov kernel function is of bounded support, which could make the diagonal matrix $\widehat{\Xi }_i(t;\beta )$ not invertible. If this happens, we replace $\widehat{\lambda }_{0k}(t)$ with the average of values at the non-zero locations.

We consider two types of simulations:

1. $\beta _k$s are the same for different failure type, i.e. $\beta _1=\ldots =\beta _K=\beta$.
2. $\beta _k$ varies across failure type.

The auxiliary covariate $A_k$ is generated from $X_k$ via

$$\begin{aligned} A_k = X_k + \epsilon _k, \end{aligned}$$

where $\epsilon _k$ follows a normal distribution $N(0,\sigma ^2)$, the positive parameter $\sigma$ controls the strength of association between $A_k$ and $X_k$. Each simulation is repeated 1000 times.

### Simulation study (1)

In the first simulation, we set the true parameter $\beta =(\beta ^{(1)},\beta ^{(2)})^{\mathrm{T}}=(0.693,-0.2)^{\mathrm{T}}$, validation proportion $\rho _k=0.5$, association parameter $\sigma =0.1$. The number of independent clusters is $n=200$, with $K=4$ or 8 failure types in each cluster.

Tables 1, 2 and 3 demonstrate the simulation results for estimates of parameter $$\beta $$ for each method under different censoring rates 10%, 40% and 80%. The sample mean and sample standard deviation of the 1000 estimates, the average of estimated standard errors and the coverage rate of the 95% confidence intervals for the true parameter are listed in the Est, SD, SE and CR columns, respectively. RE, the ratio of the empirical variance of $$\widehat{\beta }_I$$ to that of $$\widehat{\beta }_{QE}$$ or $$\widehat{\beta }_{QA}$$, is the estimated relative efficiency of estimated QIF estimators relative to $$\widehat{\beta }_I$$. We summarize the results as follows: (i) The estimates of all the methods are all approximately unbiased. Moreover, the estimators of the asymptotic standard errors are approximately equal to the empirical standard deviations. The corresponding 95% confidence intervals calculated by the estimated standard errors provide reasonable coverage rates. This suggests that the estimates of asymptotic standard errors for all methods work well. (ii) For each considered scenario, the estimator $$\widehat{\beta }_I$$ using auxiliary information is more efficient than the estimators $$\widetilde{\beta }_{QEV}$$ and $$\widetilde{\beta }_{QAV}$$ using validation set only. However, $$\widehat{\beta }_I$$ loses efficiency when the degree of correlation within a cluster becomes stronger. (iii) As *K* increases, the empirical standard deviations (SD) of all the estimators decrease. That is naturally because of the increase in the total amount of data. (iv) As $$\theta $$ decreases, the efficiency gain of estimated QIF estimators relative to $$\widehat{\beta }_I$$ increases. From Table 1, estimated QIF estimators are more efficient than the other estimators for all combinations of $$\theta $$ and *K*. We also observe the same trend from Table 2. From Table 3, however, the estimated QIF estimators are less efficient than $$\widehat{\beta }_I$$, although REs are very close to 1, in several cases due to the reduction of correlation when censoring rate is 80%. Furthermore, as expected, $$\widehat{\beta }_{QE}$$ with correct working correlation is always more efficient than $$\widehat{\beta }_{QA}$$ with misspecified working correlation. (v) The validation proportion of the incomplete covariate has effect on the values of RE, especially for the first parameter. For example, when $$K = 4 $$ and 10% censoring, the REs of $$\widehat{\beta }_{QE}$$ relative to $$\widehat{\beta }_I$$ for $$\widehat{\beta }^{(1)}$$ decrease from (3.34, 2.26, 1.21) to (2.85, 2.03, 1.18) , when validation proportion decreases from 0.5 to 0.3 (results not shown). However, when we increased *n*, not only the CRs but also the REs increased.

**Table 1:** Simulation results for common effect size across failure type: $\beta =(\beta ^{(1)},\beta ^{(2)})^{\mathrm{T}}=(0.693, -0.2)^{\mathrm{T}}$ under the censoring rate 10%

*K*	$\theta$	Method	$\widehat{\beta }^{(1)}$					$\widehat{\beta }^{(2)}$				
			Est	SD	SE	CR	RE	Est	SD	SE	CR	RE
4	0.25	$\widetilde{\beta }_{QEV}$	0.686	0.164	0.163	0.952	–	- 0.202	0.098	0.091	0.926	–
		$\widetilde{\beta }_{QAV}$	0.682	0.171	0.169	0.948	–	- 0.205	0.104	0.095	0.917	–
		$\widehat{\beta }_I$	0.675	0.143	0.141	0.945	–	- 0.203	0.077	0.075	0.938	–
		$\widehat{\beta }_{QE}$	0.684	0.078	0.078	0.942	3.34	- 0.200	0.039	0.038	0.936	3.93
		$\widehat{\beta }_{QA}$	0.680	0.089	0.088	0.933	2.62	- 0.201	0.047	0.045	0.941	2.65
	0.5	$\widetilde{\beta }_{QEV}$	0.683	0.166	0.167	0.955	–	- 0.202	0.103	0.094	0.925	–
		$\widetilde{\beta }_{QAV}$	0.679	0.174	0.173	0.946	–	- 0.205	0.107	0.097	0.926	–
		$\widehat{\beta }_I$	0.673	0.142	0.139	0.943	–	- 0.202	0.077	0.075	0.941	–
		$\widehat{\beta }_{QE}$	0.681	0.094	0.091	0.935	2.26	- 0.199	0.048	0.047	0.940	2.55
		$\widehat{\beta }_{QA}$	0.676	0.103	0.101	0.930	1.91	- 0.201	0.055	0.053	0.945	1.97
	2	$\widetilde{\beta }_{QEV}$	0.678	0.184	0.179	0.939	–	- 0.203	0.111	0.102	0.932	–
		$\widetilde{\beta }_{QAV}$	0.676	0.188	0.181	0.924	–	- 0.204	0.112	0.103	0.927	–
		$\widehat{\beta }_I$	0.673	0.139	0.137	0.946	–	- 0.201	0.078	0.075	0.938	–
		$\widehat{\beta }_{QE}$	0.675	0.127	0.123	0.938	1.21	- 0.200	0.069	0.067	0.941	1.24
		$\widehat{\beta }_{QA}$	0.672	0.133	0.127	0.928	1.11	- 0.201	0.072	0.069	0.935	1.15
8	0.25	$\widetilde{\beta }_{QEV}$	0.699	0.122	0.113	0.939	–	-0.202	0.066	0.062	0.933	–
		$\widetilde{\beta }_{QAV}$	0.697	0.130	0.121	0.929	–	-0.201	0.070	0.067	0.936	–
		$\widehat{\beta }_I$	0.683	0.106	0.103	0.941	–	-0.201	0.054	0.054	0.949	–
		$\widehat{\beta }_{QE}$	0.686	0.061	0.059	0.938	3.05	-0.200	0.027	0.026	0.942	4.12
		$\widehat{\beta }_{QA}$	0.687	0.068	0.066	0.940	2.38	-0.200	0.031	0.031	0.949	2.96
	0.5	$\widetilde{\beta }_{QEV}$	0.698	0.126	0.116	0.927	–	-0.200	0.069	0.064	0.923	–
		$\widetilde{\beta }_{QAV}$	0.695	0.133	0.123	0.935	–	-0.200	0.072	0.068	0.931	–
		$\widehat{\beta }_I$	0.684	0.103	0.101	0.945	–	-0.201	0.054	0.054	0.950	–
		$\widehat{\beta }_{QE}$	0.688	0.068	0.065	0.931	2.31	-0.200	0.032	0.032	0.946	2.89
		$\widehat{\beta }_{QA}$	0.687	0.077	0.073	0.929	1.80	-0.200	0.037	0.037	0.939	2.17
	2	$\widetilde{\beta }_{QEV}$	0.698	0.135	0.125	0.922	–	-0.199	0.074	0.071	0.941	–
		$\widetilde{\beta }_{QAV}$	0.694	0.139	0.128	0.918	–	-0.200	0.075	0.073	0.945	–
		$\widehat{\beta }_I$	0.687	0.099	0.098	0.935	–	-0.200	0.051	0.053	0.949	–
		$\widehat{\beta }_{QE}$	0.690	0.087	0.084	0.938	1.29	-0.199	0.044	0.045	0.948	1.37
		$\widehat{\beta }_{QA}$	0.687	0.094	0.090	0.931	1.13	-0.200	0.048	0.048	0.944	1.16

$\widetilde{\beta }_{QEV}$ is the estimator of the QIF method with exchangeable working correlation based only on the validation set, while $\widetilde{\beta }_{QAV}$ is the one with AR(1) working correlation. $\widehat{\beta }_I$ is the EPPL estimator using the independent structure. $\widehat{\beta }_{QE}$ and $\widehat{\beta }_{QA}$ are the estimators of the proposed estimated QIF method with exchangeable and AR(1) working correlation, respectively

**Table 2:** Simulation results for common effect size across failure type: $\beta =(\beta ^{(1)},\beta ^{(2)})^{\mathrm{T}}=(0.693, -0.2)^{\mathrm{T}}$ under the censoring rate 40%

*K*	$\theta$	Method	$\widehat{\beta }^{(1)}$					$\widehat{\beta }^{(2)}$				
			Est	SD	SE	CR	RE	Est	SD	SE	CR	RE
4	0.25	$\widetilde{\beta }_{QEV}$	0.679	0.213	0.206	0.936	–	- 0.205	0.121	0.117	0.946	–
		$\widetilde{\beta }_{QAV}$	0.676	0.222	0.213	0.932	–	- 0.206	0.124	0.121	0.950	–
		$\widehat{\beta }_I$	0.681	0.177	0.169	0.936	–	- 0.205	0.093	0.092	0.950	–
		$\widehat{\beta }_{QE}$	0.687	0.120	0.116	0.941	2.18	- 0.201	0.062	0.061	0.946	2.20
		$\widehat{\beta }_{QA}$	0.682	0.131	0.127	0.934	1.82	- 0.202	0.069	0.068	0.949	1.79
	0.5	$\widetilde{\beta }_{QEV}$	0.673	0.218	0.212	0.935	–	- 0.206	0.127	0.120	0.941	–
		$\widetilde{\beta }_{QAV}$	0.670	0.224	0.217	0.934	–	- 0.207	0.129	0.123	0.940	–
		$\widehat{\beta }_I$	0.680	0.174	0.168	0.939	–	- 0.204	0.091	0.092	0.954	–
		$\widehat{\beta }_{QE}$	0.685	0.132	0.131	0.944	1.73	- 0.202	0.070	0.070	0.952	1.66
		$\widehat{\beta }_{QA}$	0.679	0.143	0.140	0.935	1.48	- 0.203	0.076	0.076	0.954	1.45
	2	$\widetilde{\beta }_{QEV}$	0.668	0.225	0.221	0.939	–	- 0.204	0.134	0.127	0.944	–
		$\widetilde{\beta }_{QAV}$	0.666	0.226	0.222	0.934	–	- 0.205	0.134	0.127	0.937	–
		$\widehat{\beta }_I$	0.679	0.166	0.166	0.942	–	- 0.202	0.092	0.092	0.954	–
		$\widehat{\beta }_{QE}$	0.678	0.156	0.157	0.940	1.14	- 0.202	0.088	0.086	0.950	1.11
		$\widehat{\beta }_{QA}$	0.675	0.161	0.160	0.940	1.07	- 0.203	0.090	0.088	0.947	1.05
8	0.25	$\widetilde{\beta }_{QEV}$	0.702	0.152	0.143	0.929	–	- 0.201	0.084	0.080	0.944	–
		$\widetilde{\beta }_{QAV}$	0.701	0.159	0.152	0.940	–	- 0.201	0.088	0.085	0.944	–
		$\widehat{\beta }_I$	0.695	0.122	0.123	0.948	–	- 0.203	0.066	0.066	0.947	–
		$\widehat{\beta }_{QE}$	0.696	0.085	0.082	0.941	2.08	- 0.201	0.042	0.041	0.944	2.39
		$\widehat{\beta }_{QA}$	0.697	0.094	0.092	0.942	1.68	- 0.201	0.048	0.048	0.944	1.85
	0.5	$\widetilde{\beta }_{QEV}$	0.706	0.157	0.147	0.934	–	- 0.200	0.087	0.083	0.945	–
		$\widetilde{\beta }_{QAV}$	0.702	0.163	0.154	0.948	–	- 0.200	0.090	0.087	0.950	–
		$\widehat{\beta }_I$	0.697	0.120	0.121	0.946	–	- 0.202	0.066	0.065	0.951	–
		$\widehat{\beta }_{QE}$	0.700	0.092	0.090	0.945	1.69	- 0.200	0.048	0.047	0.955	1.85
		$\widehat{\beta }_{QA}$	0.699	0.102	0.099	0.938	1.39	- 0.200	0.054	0.053	0.950	1.48
	2	$\widetilde{\beta }_{QEV}$	0.700	0.165	0.155	0.939	–	- 0.199	0.089	0.088	0.947	–
		$\widetilde{\beta }_{QAV}$	0.697	0.168	0.158	0.934	–	- 0.200	0.091	0.090	0.945	–
		$\widehat{\beta }_I$	0.695	0.120	0.118	0.949	–	- 0.200	0.063	0.065	0.950	–
		$\widehat{\beta }_{QE}$	0.698	0.111	0.108	0.943	1.16	- 0.199	0.058	0.059	0.946	1.19
		$\widehat{\beta }_{QA}$	0.695	0.116	0.113	0.950	1.07	- 0.201	0.062	0.062	0.950	1.04

See Table 1

**Table 3:** Simulation results for common effect size across failure type: $\beta =(\beta ^{(1)},\beta ^{(2)})^{\mathrm{T}}=(0.693, -0.2)^{\mathrm{T}}$ under the censoring rate 80%

*K*	$\theta$	Method	$\widehat{\beta }^{(1)}$					$\widehat{\beta }^{(2)}$				
			Est	SD	SE	CR	RE	Est	SD	SE	CR	RE
4	0.25	$\widetilde{\beta }_{QEV}$	0.655	0.412	0.374	0.924	–	- 0.216	0.250	0.215	0.921	–
		$\widetilde{\beta }_{QAV}$	0.649	0.414	0.379	0.928	–	- 0.216	0.244	0.219	0.923	–
		$\widehat{\beta }_I$	0.684	0.308	0.288	0.939	–	- 0.198	0.164	0.159	0.941	–
		$\widehat{\beta }_{QE}$	0.695	0.264	0.248	0.936	1.36	- 0.199	0.147	0.136	0.935	1.24
		$\widehat{\beta }_{QA}$	0.685	0.277	0.259	0.943	1.23	- 0.200	0.153	0.143	0.936	1.15
	0.5	$\widetilde{\beta }_{QEV}$	0.652	0.435	0.381	0.929	–	- 0.211	0.241	0.221	0.938	–
		$\widetilde{\beta }_{QAV}$	0.651	0.412	0.384	0.933	–	- 0.209	0.238	0.222	0.939	–
		$\widehat{\beta }_I$	0.689	0.304	0.287	0.939	–	- 0.196	0.160	0.159	0.946	–
		$\widehat{\beta }_{QE}$	0.691	0.282	0.266	0.938	1.17	- 0.199	0.155	0.147	0.940	1.07
		$\widehat{\beta }_{QA}$	0.682	0.290	0.273	0.938	1.10	- 0.200	0.156	0.151	0.945	1.06
	2	$\widetilde{\beta }_{QEV}$	0.658	0.432	0.385	0.918	–	- 0.210	0.248	0.224	0.932	–
		$\widetilde{\beta }_{QAV}$	0.660	0.417	0.386	0.915	–	- 0.210	0.250	0.224	0.936	–
		$\widehat{\beta }_I$	0.698	0.296	0.287	0.942	–	- 0.195	0.165	0.159	0.945	–
		$\widehat{\beta }_{QE}$	0.685	0.298	0.282	0.938	0.99	- 0.202	0.167	0.157	0.936	0.98
		$\widehat{\beta }_{QA}$	0.683	0.299	0.283	0.934	0.98	- 0.202	0.167	0.157	0.931	0.97
8	0.25	$\widetilde{\beta }_{QEV}$	0.705	0.269	0.260	0.937	–	- 0.203	0.147	0.148	0.953	–
		$\widetilde{\beta }_{QAV}$	0.696	0.284	0.269	0.949	–	- 0.205	0.149	0.154	0.961	–
		$\widehat{\beta }_I$	0.706	0.214	0.206	0.937	–	- 0.205	0.111	0.113	0.953	–
		$\widehat{\beta }_{QE}$	0.710	0.178	0.169	0.939	1.44	- 0.204	0.093	0.092	0.942	1.43
		$\widehat{\beta }_{QA}$	0.706	0.195	0.183	0.928	1.20	- 0.206	0.100	0.100	0.956	1.23
	0.5	$\widetilde{\beta }_{QEV}$	0.699	0.281	0.266	0.934	–	- 0.202	0.151	0.153	0.949	–
		$\widetilde{\beta }_{QAV}$	0.692	0.288	0.271	0.930	–	- 0.204	0.153	0.156	0.947	–
		$\widehat{\beta }_I$	0.707	0.204	0.204	0.937	–	- 0.202	0.111	0.112	0.954	–
		$\widehat{\beta }_{QE}$	0.709	0.188	0.183	0.938	1.19	- 0.202	0.100	0.101	0.954	1.22
		$\widehat{\beta }_{QA}$	0.705	0.199	0.193	0.929	1.06	- 0.204	0.106	0.106	0.949	1.10
	2	$\widetilde{\beta }_{QEV}$	0.684	0.291	0.273	0.932	–	- 0.205	0.164	0.157	0.937	–
		$\widetilde{\beta }_{QAV}$	0.681	0.290	0.273	0.929	–	- 0.204	0.161	0.157	0.942	–
		$\widehat{\beta }_I$	0.705	0.207	0.203	0.943	–	- 0.203	0.112	0.112	0.954	–
		$\widehat{\beta }_{QE}$	0.702	0.207	0.198	0.942	1.00	- 0.205	0.111	0.109	0.947	1.02
		$\widehat{\beta }_{QA}$	0.700	0.209	0.200	0.944	0.98	- 0.206	0.112	0.110	0.947	0.99

See Table 1

### Simulation study (2)

In practical studies, one may be interested in the failure-type-specific model

$$\begin{aligned} \lambda _{ik}(t;\widetilde{Z}_{ik}(t))=\lambda _{0k}(t) \exp \{\beta _k^{\mathrm{T}}\widetilde{Z}_{ik}(t)\}, \end{aligned}$$

which allows the regression parameters varying with the failure type. We simulate $K=2$ failure types in each cluster, the true parameter $\beta _{1}=(\beta _1^{(1)},\beta _1^{(2)})^{\mathrm{T}} =(0.693,-0.2)^{\mathrm{T}}$ and $\beta _{2}=(\beta _2^{(1)}, \beta _2^{(2)})^{\mathrm{T}}=(0.5,-0.262)^{\mathrm{T}}$. Since the cluster size is 2, we only need to consider the exchangeable working correlation structure. Consider three settings of *n* and censoring rate (CE), (*n*, CE)$\,=\,(300,10\%), (300,40\%), (700,80\%)$. The simulation results are shown in Table 4. From this table, we can observe similar results as in Simulation Study (1).

**Table 4:** Simulation results for varying effect size across failure type: $\beta _1=(\beta _1^{(1)},\beta _1^{(2)})^{\mathrm{T}} =(0.693, -0.2)^{\mathrm{T}}, \beta _2=(\beta _2^{(1)}, \beta _2^{(2)})^{\mathrm{T}}=(0.5, -0.262)^{\mathrm{T}}$

$\theta$	Method	$\widehat{\beta }_1^{(1)}$					$\widehat{\beta }_1^{(2)}$					$\widehat{\beta }_2^{(1)}$					$\widehat{\beta }_2^{(2)}$				
		Est	SD	SE	CR	RE	Est	SD	SE	CR	RE	Est	SD	SE	CR	RE	Est	SD	SE	CR	RE
		$n=300$, censoring rate 10%																			
0.25	$\widetilde{\beta }_{QEV}$	0.696	0.294	0.272	0.941	–	- 0.191	0.170	0.155	0.931	–	0.477	0.296	0.270	0.929	–	- 0.263	0.172	0.156	0.928	–
	$\widehat{\beta }_I$	0.693	0.229	0.222	0.940	–	- 0.194	0.121	0.123	0.948	–	0.495	0.226	0.222	0.943	–	- 0.257	0.127	0.124	0.946	–
	$\widehat{\beta }_{QE}$	0.681	0.139	0.130	0.934	2.69	- 0.200	0.073	0.069	0.930	2.75	0.493	0.139	0.128	0.915	2.62	- 0.263	0.070	0.069	0.949	3.31
0.5	$\widetilde{\beta }_{QEV}$	0.694	0.301	0.278	0.934	–	- 0.190	0.172	0.158	0.926	–	0.484	0.302	0.277	0.929	–	- 0.263	0.176	0.160	0.925	–
	$\widehat{\beta }_I$	0.693	0.229	0.222	0.941	–	- 0.194	0.121	0.123	0.948	–	0.498	0.226	0.222	0.936	–	- 0.258	0.127	0.124	0.946	–
	$\widehat{\beta }_{QE}$	0.682	0.170	0.158	0.937	1.81	- 0.198	0.090	0.085	0.932	1.82	0.495	0.170	0.157	0.930	1.76	- 0.263	0.087	0.085	0.947	2.13
2	$\widetilde{\beta }_{QEV}$	0.688	0.315	0.291	0.938	–	- 0.189	0.179	0.166	0.932	–	0.482	0.315	0.291	0.921	–	- 0.267	0.184	0.168	0.926	–
	$\widehat{\beta }_I$	0.693	0.229	0.222	0.941	–	- 0.194	0.121	0.123	0.948	–	0.502	0.225	0.222	0.950	–	- 0.262	0.123	0.124	0.961	–
	$\widehat{\beta }_{QE}$	0.677	0.223	0.206	0.932	1.05	- 0.195	0.117	0.113	0.937	1.08	0.490	0.220	0.206	0.938	1.05	- 0.266	0.117	0.114	0.943	1.10
		$n=300$, censoring rate 40%																			
0.25	$\widetilde{\beta }_{QEV}$	0.687	0.368	0.342	0.933	–	- 0.202	0.203	0.195	0.946	–	0.449	0.375	0.349	0.919	–	- 0.270	0.220	0.202	0.934	–
	$\widehat{\beta }_I$	0.702	0.273	0.269	0.953	–	- 0.195	0.142	0.148	0.956	–	0.490	0.265	0.274	0.953	–	- 0.260	0.155	0.153	0.950	–
	$\widehat{\beta }_{QE}$	0.684	0.213	0.203	0.945	1.64	- 0.204	0.113	0.110	0.937	1.59	0.477	0.210	0.206	0.936	1.58	- 0.266	0.119	0.114	0.931	1.71
0.5	$\widetilde{\beta }_{QEV}$	0.684	0.378	0.348	0.931	–	- 0.199	0.209	0.199	0.940	–	0.461	0.383	0.356	0.927	–	- 0.272	0.219	0.206	0.939	–
	$\widehat{\beta }_I$	0.702	0.273	0.269	0.953	–	- 0.195	0.142	0.148	0.956	–	0.498	0.271	0.274	0.948	–	- 0.260	0.155	0.153	0.948	–
	$\widehat{\beta }_{QE}$	0.684	0.237	0.226	0.941	1.32	- 0.199	0.126	0.124	0.940	1.28	0.483	0.238	0.231	0.947	1.30	- 0.266	0.130	0.128	0.947	1.44
2	$\widetilde{\beta }_{QEV}$	0.680	0.387	0.357	0.933	–	- 0.200	0.214	0.205	0.935	–	0.481	0.411	0.366	0.913	–	- 0.276	0.225	0.212	0.932	–
	$\widehat{\beta }_I$	0.702	0.273	0.269	0.953	–	- 0.195	0.142	0.148	0.956	–	0.508	0.282	0.275	0.939	–	- 0.260	0.152	0.153	0.950	–
	$\widehat{\beta }_{QE}$	0.683	0.271	0.259	0.942	1.01	- 0.200	0.142	0.142	0.950	1.01	0.491	0.282	0.264	0.932	1.00	- 0.268	0.148	0.147	0.946	1.05
		$n=700$, censoring rate 80%																			
0.25	$\widetilde{\beta }_{QEV}$	0.672	0.404	0.397	0.948	–	- 0.213	0.241	0.229	0.947	–	0.477	0.471	0.418	0.912	–	- 0.283	0.259	0.243	0.938	–
	$\widehat{\beta }_I$	0.701	0.311	0.299	0.940	–	- 0.200	0.164	0.166	0.955	–	0.506	0.323	0.314	0.942	–	- 0.262	0.169	0.176	0.958	–
	$\widehat{\beta }_{QE}$	0.686	0.283	0.272	0.936	1.20	- 0.206	0.150	0.151	0.948	1.18	0.494	0.305	0.286	0.938	1.12	- 0.270	0.162	0.160	0.948	1.09
0.5	$\widetilde{\beta }_{QEV}$	0.670	0.412	0.401	0.938	–	- 0.213	0.245	0.232	0.952	–	0.473	0.476	0.422	0.922	–	- 0.283	0.267	0.246	0.934	–
	$\widehat{\beta }_I$	0.701	0.311	0.299	0.940	–	- 0.200	0.164	0.166	0.955	–	0.510	0.328	0.314	0.943	–	- 0.267	0.172	0.176	0.957	–
	$\widehat{\beta }_{QE}$	0.683	0.299	0.286	0.942	1.08	- 0.207	0.160	0.159	0.950	1.04	0.494	0.327	0.301	0.931	1.01	- 0.276	0.172	0.168	0.947	1.00
2	$\widetilde{\beta }_{QEV}$	0.672	0.420	0.405	0.933	–	- 0.215	0.247	0.234	0.947	–	0.474	0.458	0.425	0.935	–	- 0.278	0.274	0.248	0.934	–
	$\widehat{\beta }_I$	0.701	0.311	0.299	0.940	–	- 0.200	0.164	0.166	0.955	–	0.521	0.316	0.314	0.948	–	- 0.263	0.183	0.176	0.948	–
	$\widehat{\beta }_{QE}$	0.687	0.313	0.295	0.930	0.99	- 0.207	0.165	0.165	0.947	0.99	0.499	0.324	0.310	0.941	0.96	- 0.271	0.187	0.174	0.940	0.96

The cluster size $K=2$, validation proportion $\rho _k=0.5$, association parameter $\sigma =0.1$

### Performance of inference method

**Fig. 1:**
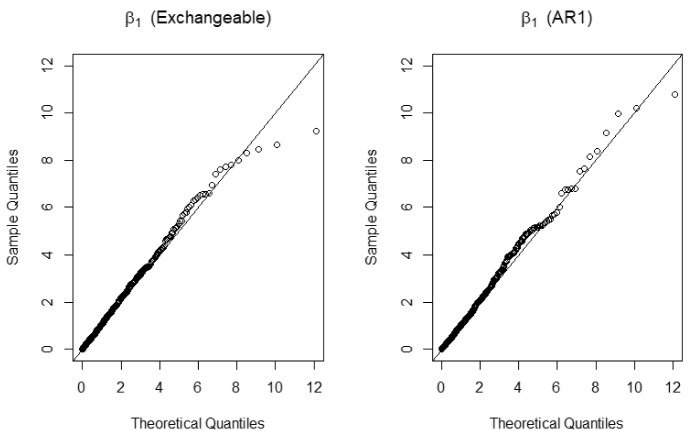
Q–Q plot for the test statistic versus $\chi _1^2$ under $H_0$ for 1000 replications

**Fig. 2:**
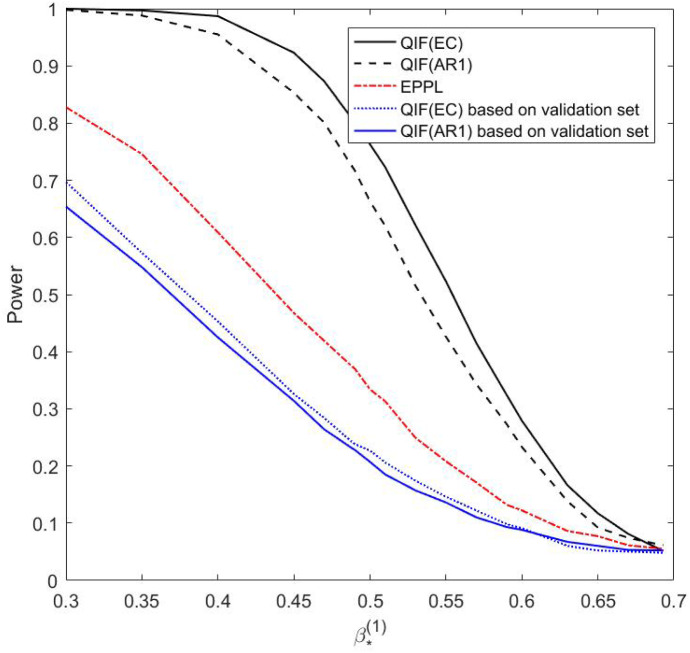
Power functions of the chi-squared test for the proposed method, the EPPL method and the QIF method under $H_0: \beta ^{(1)} =0.693, H_1: \beta ^{(1)} = \beta _{*}^{(1)}$

We also conduct simulation studies to assess the performance of the proposed chi-squared test method. The data are generated from the same model as in Simulation Study (1) with $\beta =(\beta ^{(1)}, \beta ^{(2)})^{\mathrm{T}}=(0.693,-0.2)^{\mathrm{T}}, n=200, K=4, \theta =0.25, \rho _k=0.5, \sigma =0.1$, and censoring rate is 10%. First, we are interested in testing $H_0: \beta ^{(1)} =0.693$ versus $H_1: \beta ^{(1)} \ne 0.693$. Since the dimension of $\beta ^{(1)}$ is 1, the test statistic *T* in (4.1) asymptotically follows $\chi _1^2$, where $\widetilde{\beta }^{(2)}$ is calculated by minimizing $\varvec{\widehat{Q}}_n(0.693, \beta ^{(2)})$ with exchangeable or AR(1) working correlation. Figure 1 shows Q–Q plot based on 1000 replications. It is clear that the plots indicate proximity to the $\chi _1^2$ distribution for both exchangeable and AR(1) working correlation. We also examine the power of the proposed test under $H_1: \beta ^{(1)} =\beta _{*}^{(1)}$. The powers with significance level $\alpha =0.05$ are calculated when $\beta _{*}^{(1)}$ takes different values in [0.3, 0.693]. According to the simulation results, when $\beta _{*}^{(1)} = 0.693$, i.e., the alternative hypothesis collapses into the null hypothesis, powers are 0.051 and 0.061 for exchangeable and AR(1) working correlation, respectively. It shows that the proposed chi-squared test gives the right level for testing. Figure 2 plots the power functions of the chi-squared test for the estimated QIF method and the QIF method with two different working correlations, and the EPPL method. We can observe that the power functions decrease rapidly as $\beta _{*}^{(1)}$ gets closer to the true value (0.693), but the power function for exchangeable working correlation is always larger than that for AR(1) working correlation, thus the test with correct working correlation is more powerful than the one with misspecified working correlation. Nonetheless, powers of the chi-squared test for either of the estimated QIF methods are larger than those of the other two methods. In addition, the power for the EPPL method that utilizes the auxiliary information is larger than those for QIF method based on the validation set only. Similarly, we also consider the hypothesis test that $H_0: \beta ^{(2)} = -0.2$, and $H_1: \beta ^{(2)} \ne -0.2$, and compute the powers under $H_1: \beta ^{(2)} = \beta _{*}^{(2)}$ when $\beta _{*}^{(2)}$ varies in $[-0.2, 0]$. Similar results are obtained but not presented in this paper due to space limitation.

## Analysis of SOLVD data

We apply the proposed method to Left Ventricular Dysfunction (SOLVD 1991) study in this section. The SOLVD study was a randomized, double-masked, placebo-controlled trial between 1986 and 1991. The trial had a three-year recruitment and a two-year follow-up. The basic inclusion criteria for the prevention trial were: age between 21 and 80 years, inclusive, no overt symptoms of congestive heart failure, and left ventricular EF less than 35%. EF is a number between 0 and 100 that measures the efficiency of the heart in ejecting blood. A total of 4228 patients with asymptomatic left ventricular dysfunction were randomly assigned to receive either enalapril or placebo at one of the 83 hospitals linked to 23 centers in the United States, Canada, and Belgium. Liu et al. (2009) and Liu et al. (2010) have analyzed this data without considering the intra-cluster correlation.

The primary clinical issues of interest are the effects of covariates on the risk of heart failure and on the first nonfatal myocardial infarction (MI) after adjusting for the confounding variables. The covariates of interest are ejection fraction, patient’s gender (SEX), which is coded 1 for male and 0 for female, treatment (TRT), which is coded 1 for enalapril and 0 for placebo, and patient’s age (AGE), which is measured in years. In the SOLVD study, the covariates SEX, TRT and AGE were recorded for almost all of the patients, but only 108 among the total of 4228 patients have their ejection fraction accurately measured using a standardized radionucleotide technique (LVEF). A related nonstandardized measure (EF) was ascertained for all the patients. Therefore, the nonstandardized measure (EF) is a surrogate measure for the standardized measure (LVEF) in this case.

In terms of the notation in the previous sections, we set $X_{ik}=LVEF_{ik}, A_{ik}=EF_{ik}, Z_{ik}=(TRT_{ik}, SEX_{ik}, AGE_{ik})^{\mathrm{T}}$, where *k* denotes failure type with $k=1$ for heart failure and $k=2$ for nonfatal MI and *i* denotes the patient with $i=1, \ldots , 4228$. Let $\beta _k = (\beta _{1k}, \beta _{2k}^{\mathrm{T}})^{\mathrm{T}}$ be the unknown regression coefficients, we fit the following marginal hazards model to the SOLVD data:

$$\begin{aligned} \lambda _{ik}(t; X_{ik}, Z_{ik}) = \lambda _{0k}(t) \exp (\beta _{1k}X_{ik} + \beta _{2k}^{\mathrm{T}} Z_{ik}). \end{aligned}$$

Since the primary covariate LVEF is continuous and severely incomplete, we need to estimate the induced relative risk function using the validation set. Furthermore, Liu et al. (2009) found that, given EF, the LVEF is conditionally independent of the rest of the covariates, thus $\psi _{ik}(t;\beta )$ can be estimated through (2.8) with $A_{ik}^{*} = A_{ik}$.

Table 5 presents the data analysis results for two methods, the proposed estimated QIF method, and the EPPL method which ignores the intra-cluster correlation between the failure times. It can be seen that the parameter estimates from two methods are close but the proposed estimated QIF method have smaller standard errors. To test whether or not the covariates have significant effects on the times of heart failure and nonfatal MI, we calculate p-values from both the two-sided Z-test and the chi-squared test for the two methods. The results indicate that, at 0.05 significance level, all of the covariates are statistically significant for heart failure by the proposed method, while SEX is not significant from the EPPL method. From both methods, only TRT is significant for the risk of nonfatal MI. By the proposed method, the risk of heart failure decreases by 3.92% (95% CI [1.83%, 5.97%]) with 1% increase in LVEF, the risk increases by 2.63% (95% CI [1.43%, 3.85%]) per year increase in age, males have about 29.46% (95% CI [7.19%, 46.39%]) lower risk for heart failure than females, and enalapril reduces the risk by 33.77% (95% CI [20.52%, 44.80%]).

**Table 5:** SOLVD data analysis results

Covariate	Proposed method				EPPL method			
	Coef	SE	*P* value		Coef	SE	*P* value	
			Z-test	$\chi ^2$-test			Z-test	$\chi ^2$-test
For heart failure								
LVEF	- 0.040	0.011	< 0.001	< 0.001	- 0.045	0.012	< 0.001	< 0.001
TRT	- 0.412	0.093	< 0.001	< 0.001	- 0.454	0.106	< 0.001	< 0.001
SEX	- 0.349	0.140	0.013	< 0.001	- 0.318	0.165	0.054	0.072
AGE	0.026	0.006	< 0.001	< 0.001	0.023	0.012	0.045	0.009
For nonfatal MI								
LVEF	0.006	0.011	0.566	0.534	0.023	0.015	0.111	0.102
TRT	- 0.433	0.116	< 0.001	< 0.001	- 0.391	0.131	0.003	0.002
SEX	0.084	0.196	0.669	0.294	0.048	0.214	0.822	0.815
AGE	0.006	0.006	0.275	0.715	0.004	0.008	0.651	0.597

Proposed method is referred to as the estimated QIF method with exchangeable working correlation. EPPL method is the method which uses the independent structure

**Fig. 3:**
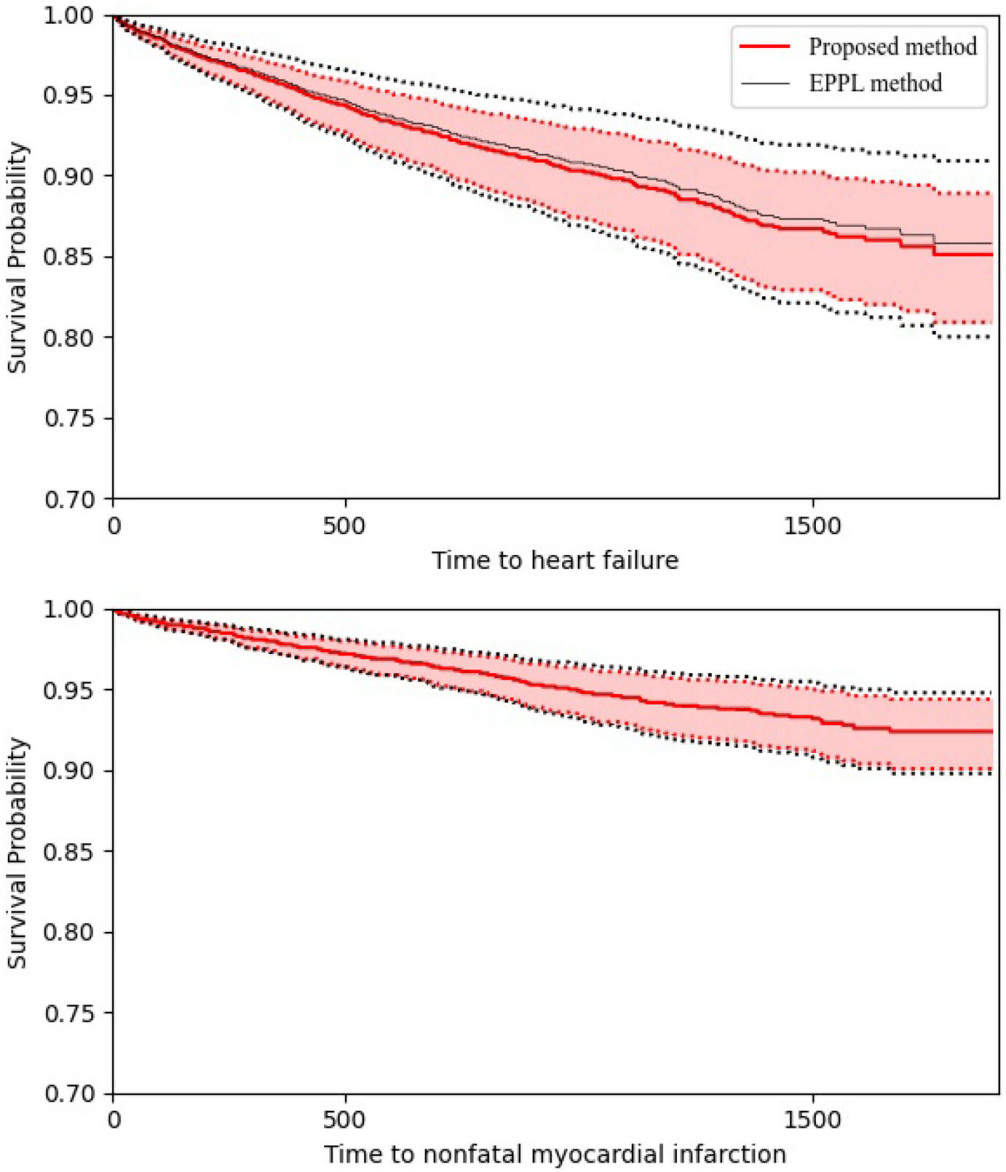
Survival curves by the proposed method (bold curve) and the EPPL method (thin curve) for heart failure and nonfatal myocardial infarction of a subject with covariates $\hbox {LVEF}=28\%, \hbox {TRT}=1, \hbox {SEX}=1$ and $\hbox {AGE}=69$, along with the corresponding 95% pointwise confidence intervals (dotted curves)

To illustrate the prediction of the survival probability for a subject, Fig. 3 shows the estimated survival curves of heart failure and nonfatal MI for a 69-year-old male patient with LVEF of 28% (the median of LVEF), receiving enalapril. The survival curves by the two methods are very close, but the pointwise confidence intervals from the proposed method are narrower than those from the EPPL method.

## Concluding remarks

We proposed an estimated QIF approach for multivariate failure time data when the primary covariate is ascertained on a subset of full cohort but auxiliary information is available on the full cohort. For our proposed approach, we allowed the censoring times for different failures for a subject to be different. It is worth noting that in practice the censoring times for different failures for a subject are usually the same. This can be treated as a special situation of our general set up and the proposed method is applicable.

In this article, we consider the situation that auxiliary variable is continuous. The method is based on the kernel smoothing technique and therefore is nonparametric with respect to the association between the missing covariate and corresponding auxiliary. QIF method has advantage of incorporating intra-cluster correlation in the estimation procedure. Compared with other existing methods (e.g., Liu et al. 2010) where intra-cluster correlation is not considered, the proposed procedure can improve the estimation efficiency without requiring the specification of the correlation formula. Another advantage of proposed method is that it is easy to implement. In this work, we consider the situation when the dimension of continuous auxiliary covariates is low, further research is needed when the dimension is high.

## Appendix

For $i=1, \ldots , n$, let $\Phi _i(t; \beta , B) = \Xi _i^{1/2}(t; \beta ) B \Xi _i^{-1/2}(t; \beta )$, and $\Phi _i(t; \beta , B) = \{ \phi _{imk} \}_{m,k=1}^K$. For $k=1, \ldots , K$, some quantities are defined as follows:

$$\begin{aligned}&S_k^{d}(t; \beta ) = n^{-1} \sum \limits _{l=1}^{n} Y_{lk}(t) r_{lk}^{(d)} (t;\beta ), (d = 0, 1),\\&S_k^{d}(t; \beta , B) = n^{-1} \sum \limits _{l=1}^{n} \sum \limits _{m=1}^{K} Y_{lk}(t) R_{lm}(t;\beta ) \phi _{lmk}(t; \beta , B) (r_{lk}^{(d-2)} (t;\beta ))^{\mathrm{T}}, (d=2, 3),\\&S_k^{4}(t; \beta , B) = n^{-1} \sum \limits _{l=1}^{n} \sum \limits _{m=1}^{K} Y_{lk}(t) R_{lm}(t;\beta ) \{\partial \phi _{lmk}(t;\beta , B) /\partial \beta ^{\mathrm{T}} \} r_{lk}(t;\beta ),\\&S_k^{5}(t; \beta , B) = n^{-1} \sum \limits _{l=1}^{n} \sum \limits _{m=1}^{K} Y_{lk}(t) \{\partial R_{lm}(t;\beta ) / \partial \beta ^{\mathrm{T}} \} \phi _{lmk}(t;\beta , B) r_{lk}(t;\beta ). \end{aligned}$$

For $d = 0,\ldots , 5$, define $\widehat{S}_k^{d}$ by substituting $\widehat{r}_{lk}(t;\beta ), \widehat{R}_{lm}(t;\beta )$ and $\widehat{\phi }_{lmk}(t;\beta , B) =\widehat{\lambda }_{lm}^{1/2}(t;\beta ) B_{mk} \widehat{\lambda }_{lk}^{-1/2}(t;\beta )$ for $r_{lk}(t;\beta )$, $R_{lm}(t;\beta )$ and $\phi _{lmk}(t;\beta , B)$ in $S_k^{d}$, respectively. Furthermore, we also define

$$\begin{aligned} \varvec{E}_k(t; \beta , B)= \frac{S_k^{2}(t; \beta , B)}{S_k^{0} (t; \beta )}, \varvec{V}_k(t; \beta , B)= \frac{S_k^{3} (t; \beta , B)}{S_k^{0}(t; \beta )} - \frac{S_k^{2} (t; \beta , B)(S_k^{1}(t; \beta ))^{\mathrm{T}} }{(S_k^{0}(t; \beta )^2)} . \end{aligned}$$

We introduce some conditions to ensure the consistency and asymptotic normality of $\widehat{\beta }_Q$ and $\widehat{\beta }_I$ as follows:

1. $$\Lambda _{0k}(\tau ) = \int _0^\tau \lambda _{0k}(t) \mathrm{d}t <\infty $$, for $$k = 1,\ldots , K$$.
2. $$P\{Y_{ik}(t)=1\}>0$$, for all $$t \in [0,\tau ], i=1, \ldots , n$$.
3. There exists a compact set ${\mathscr {B}}$, containing $\beta _0$ as its interior point.
4. Multivariate kernel function $\Psi (\cdot )$ is non-negative and uniformly bounded with finite support satisfying that $\int \Psi (\varvec{u}) \mathrm{d} \varvec{u} =1$ and $\int \Psi ^2(\varvec{u}) \mathrm{d} \varvec{u} < \infty$. Furthermore, $\Psi (\cdot )$ has order $\alpha _{0}$ in the sense that $\alpha _{0} \equiv \inf \{|\alpha |>b; \int _{R^b}\varvec{u}^{\alpha } \Psi (\varvec{u}) \mathrm{d} \varvec{u} \ne 0 \}$, where *b* is the dimension of $\varvec{u}, \varvec{u}=(u_1,\ldots , u_b)$, $\alpha =(\alpha _1,\ldots , \alpha _b)$ with $\alpha _i$s being non-negative integers, $\varvec{u}^{\alpha }=u_1^{\alpha _1}\ldots u_b^{\alpha _b}$.
  For $k=1, \ldots , K$, the bandwidth matrix $\mu _k$ satisfies that $\sqrt{n}\Vert \mu _k\Vert ^{\alpha _{0}} \rightarrow 0$ and $\log n/(\sqrt{n}\Vert \mu _k\Vert ^{b}) \rightarrow 0$.
5. Let $\alpha _{0}$ be as in (C4), $h_k(\varvec{v}, \varvec{s}) = \frac{\partial ^2 H_k(1, \varvec{v}, \varvec{s})}{\partial \varvec{v} \partial \varvec{s}}$ has the $\alpha _{0}$th continuous derivative with respect to $\varvec{v}$, where $H_k(\varvec{u}, \varvec{v}, \varvec{s})$ is the joint distribution function of $(\eta _k Y_{k}(t), A_k^{*}(t), X_k(t))$ for given *t*.
6. There exist scalar, vector and matrix functions $s_k^d(t; \beta ) (d = 0, 1), s_k^d(t; \beta , B)$ $(d = 2, \ldots , 5)$, such that for all $k=1, \ldots , K$ and all constant matrix *B*,
  $$\begin{aligned} \underset{t \in [0,\tau ], \beta \in {\mathscr {B}}}{\mathrm{sup}}\Vert S_k^d-s_k^d\Vert \rightarrow 0, \end{aligned}$$
  in probability.
7. Let $s_k^d(t; \beta ) (d = 0, 1), s_k^d(t; \beta , B) (d = 2, \ldots , 5)$ be as in Condition (C6), and set $\varvec{e}_k(t;\beta , B) = s_k^{2}(t; \beta , B)/s_k^{0}(t; \beta ), \varvec{v}_k(t; \beta , B)= s_k^{3}(t; \beta , B)/s_k^{0}(t; \beta ) - s_k^{2}(t; \beta , B)(s_k^{1}(t; \beta ))^{\mathrm{T}} / (s_k^{0}(t; \beta )^2)$. Then for all $\beta \in {\mathscr {B}}$, $t \in [0,\tau ]$, and $k=1, \ldots , K$,
  $$\begin{aligned} s_k^{1}(t; \beta )= & {} \partial s_k^{0}(t; \beta ) / \partial \beta , \\ s_k^{3}(t; \beta , B)= & {} \partial s_k^{2}(t; \beta ,B) /\partial \beta -s_k^{4}(t; \beta ,B)- s_k^{5}(t; \beta ,B), \end{aligned}$$
  and $s_k^{0}(t; \beta )$ is bounded away from 0 on $[0,\tau ] \times {\mathscr {B}}$. For all basis matrix *B*, the matrix
  A.1 $$\begin{aligned} \Gamma (\beta _0, B) = - \sum _{k=1}^{K} \int _{0}^{\tau } \varvec{v}_k(t; \beta _0, B) s_k^0(t; \beta _0)\lambda _{0k}(t) \mathrm{d}t \end{aligned}$$
  is negative definite.
8. There exists a matrix function $\varvec{\omega }(\cdot , \cdot , \cdot )$, such that for any $K \times K$ constant matrices $B_1, B_2$,
  $$\begin{aligned} n^{-1} \sum _{i=1}^{n} \sum _{k=1}^{K} \int _{0}^{\tau } \varvec{\zeta }_{ik}(t;\beta , B_1) \varvec{\zeta }_{ik}^{\mathrm{T}} (t;\beta , B_2) s_k^{0}(t; \beta ) \lambda _{0k}(t) \mathrm{d}t \rightarrow \varvec{\omega }(\beta , B_1, B_2), \end{aligned}$$
  in probability uniformly for $\beta \in {\mathscr {B}}$, where
  $$\begin{aligned} \varvec{\zeta }_{ik}(t;\beta , B) = \sum _{m=1}^{K} R_{im} (t; \beta ) \phi _{imk}(t;\beta , B) - \varvec{e}_k(t; \beta , B). \end{aligned}$$
  Furthermore, for any set of basis matrices $\{B_j, j=1, \ldots , L\}$, the matrix
  A.2 $$\begin{aligned} \varvec{W}_0 = \varvec{W}(\beta _0) = \{\varvec{\omega }(\beta _0, B_j, B_{j^{'}})\}_{j, j^{'} =1}^L \end{aligned}$$
  is positive definite.
9. The baseline hazard rates $\{\lambda _{0k}(\cdot ), k=1,\ldots , K\}$ are twice continuously differentiable on $[0,\tau ]$.

### Proof of Theorem 1

**Consistency**

Following the arguments of Xue et al. (2010), one can show that $\widehat{\beta }_Q$ is consistent for $\beta _0$ provided that:

(i) $\partial \widehat{\varvec{G}}_n(\beta )/ \partial \beta ^{\mathrm{T}}$ exists and is continuous, and it converges in probability to a fixed function, say $J(\beta )$, uniformly for $\beta \in {\mathscr {B}}$;
(ii) $\widehat{\varvec{G}}_n(\beta _0) \rightarrow 0$ in probability;
(iii) $n\widehat{\varvec{W}}_n(\beta ) = \frac{1}{n} \sum _{i=1}^{n} \widehat{\varvec{g}}_i(\beta ) \widehat{\varvec{g}}_i^{\mathrm{T}}(\beta )$ converges in probability to a constant matrix $\varvec{W}(\beta )$ uniformly for $\beta \in {\mathscr {B}}$;
(iv) $n\widehat{\varvec{W}}_n(\beta _0)$ is positive definite with probability going to 1 as $n \rightarrow \infty$.

According to the first conclusion of Lemma 1 in Liu et al. (2010), under conditions (C2), (C4) and (C5), for $a=0,1,2$, we can prove that in probability

$$\begin{aligned} \underset{t\in [0,\tau ], \beta \in {\mathscr {B}}}{\sup } \Vert \widehat{\psi }_{ik}^{(a)}(t; \beta )- \psi _{ik}^{(a)}(t; \beta )\Vert \rightarrow 0. \end{aligned}$$

Since the kernel smoothed estimator $\widehat{\lambda }_{0k}(t)$ in (2.7) is a consistent estimator for $\lambda _{0k}(t)$, then, by the definitions of $\widehat{S}_{k}^{d}$ and $S_{k}^{d}$ and the remaining conditions, it follows that

$$\begin{aligned} \underset{t\in [0,\tau ], \beta \in {\mathscr {B}}}{\sup } \Vert \widehat{S}_{k}^{d}- S_{k}^{d} \Vert \rightarrow 0, \end{aligned}$$

in probability for $d=0, 1, \ldots , 5$.

Denote

$$\begin{aligned} \widehat{U}(\beta , B) = \frac{1}{n} \sum _{i=1}^{n} \int _{0}^{\tau } \varvec{\widehat{R}}_i^{\mathrm{T}}(t; \beta ) \widehat{\Xi }_i^{1/2}(t; \beta ) B \widehat{\Xi }_i^{-1/2}(t; \beta ) \widehat{\varvec{M}}_i(\mathrm{d}t; \beta ), \end{aligned}$$

then $$\widehat{\varvec{G}}_n(\beta ) = (\widehat{U}^{\mathrm{T}}(\beta , B_1), \dots , \widehat{U}^{\mathrm{T}}(\beta , B_L))^{\mathrm{T}}$$.

After simple algebraic manipulations, we obtain that

$$\begin{aligned} \widehat{U}(\beta , B)&= \frac{1}{n} \sum _{i=1}^{n} \sum _{k=1}^{K} \int _{0}^{\tau } \widehat{\varvec{\zeta }}_{ik} (t;\beta , B) \mathrm{d} N_{ik}(t)\\&=\frac{1}{n} \sum _{i=1}^{n} \sum _{k=1}^{K} \int _{0}^{\tau } \widehat{\varvec{\zeta }}_{ik} (t;\beta , B) \mathrm{d} M_{ik}(t) \\&\quad + \frac{1}{n} \sum _{i=1}^{n} \sum _{k=1}^{K} \int _{0}^{\tau } \widehat{\varvec{\zeta }}_{ik} (t;\beta , B) Y_{ik}(t)r_{ik}(t; \beta ) \lambda _{0k}(t) \mathrm{d}t, \end{aligned}$$

where $$\widehat{\varvec{\zeta }}_{ik} (t;\beta , B)=\sum _{m=1}^{K} \widehat{R}_{im}(t; \beta ) \widehat{\phi }_{imk}(t;\beta , B) -{\widehat{S}_k^{2}(t; \beta , B)}/{\widehat{S}_k^{0}(t; \beta )}$$, and

7.3 $$\begin{aligned} \frac{\partial \widehat{U}(\beta , B) }{\partial \beta ^{\mathrm{T}}}&= \frac{1}{n} \sum _{i=1}^{n} \sum _{k=1}^{K} \int _{0}^{\tau } \frac{\partial \widehat{\varvec{\zeta }}_{ik} (t;\beta , B)}{\partial \beta ^{\mathrm{T}}} \mathrm{d}M_{ik}(t)\nonumber \\&\quad + \frac{1}{n} \sum _{i=1}^{n} \sum _{k=1}^{K} \int _{0}^{\tau } \frac{\partial \widehat{\varvec{\zeta }}_{ik} (t;\beta , B)}{\partial \beta ^{\mathrm{T}}} Y_{ik}(t)r_{ik}(t; \beta )\lambda _{0k}(t) \mathrm{d}t, \end{aligned}$$

with

$$\begin{aligned} \frac{\partial \widehat{\varvec{\zeta }}_{ik} (t;\beta , B)}{\partial \beta ^{\mathrm{T}}}&=\sum _{m=1}^{K} \frac{\partial \widehat{R}_{im}(t; \beta ) \widehat{\phi }_{imk} (t;\beta , B)}{\partial \beta ^{\mathrm{T}}} + \frac{\widehat{S}_k^{2}(t; \beta , B) (\widehat{S}_k^{1}(t; \beta ))^{\mathrm{T}}}{(\widehat{S}_k^{0}(t; \beta ))^2}\\&\quad -\frac{\widehat{S}_k^{3}(t; \beta , B)+ \widehat{S}_k^{4}(t; \beta , B) + \widehat{S}_k^{5}(t; \beta , B) }{\widehat{S}_k^{0}(t; \beta )}. \end{aligned}$$

Clearly, $\partial \widehat{U}(\beta , B) /\partial \beta ^{\mathrm{T}}$ is continuous. For any constant matrix *B*, the first term on the right hand side of (7.3) is a local square integrable martingale, hence by Lenglart inequality, we can show that it converges to zero in probability, uniformly for $\beta \in {\mathscr {B}}$. Let $\Gamma (\beta , B)$ denote the uniform convergence limit of the second term, we can show that

$$\begin{aligned} \frac{\partial \widehat{U}(\beta , B) }{\partial \beta ^{\mathrm{T}}} \rightarrow \Gamma (\beta , B) = -\sum _{k=1}^{K} \int _{0}^{\tau } \varvec{v}_k(t; \beta ,B) s_k^0(t; \beta )\lambda _{0k}(t) \mathrm{d}t \end{aligned}$$

in probability, uniformly in $\beta \in {\mathscr {B}}$. Thus, $\partial \varvec{G}_n(\beta )/\partial \beta ^{\mathrm{T}}$ converges to

$$\begin{aligned} J(\beta ) = \left( \begin{array}{c} \Gamma (\beta , B_1)\\ \vdots \\ \Gamma (\beta , B_L) \end{array}\right) \end{aligned}$$

in probability, uniformly for $\beta \in {\mathscr {B}}$. Therefore, (i) is satisfied.

Denote

$$\begin{aligned} \widehat{U}_1(\beta , B) = \frac{1}{n} \sum _{k=1}^{K} \sum _{i=1}^{n} \int _{0}^{\tau } \widehat{\varvec{\zeta }}_{ik} (t;\beta , B) \mathrm{d} M_{ik}(t), \end{aligned}$$

and

$$\begin{aligned} \widehat{U}_2(\beta , B) = \frac{1}{n} \sum _{k=1}^{K} \sum _{i=1}^{n} \int _{0}^{\tau } \widehat{\varvec{\zeta }}_{ik} (t;\beta , B) Y_{ik}(t) r_{ik}(t; \beta ) \lambda _{0k}(t) \mathrm{d}t. \end{aligned}$$

When $\beta = \beta _0$, by Lenglart inequality, we can show that

$$\begin{aligned} \sqrt{n}\widehat{U}_1(\beta _0, B) = \frac{1}{\sqrt{n}} \sum _{k=1}^{K} \sum _{i=1}^{n} \int _{0}^{\tau } \varvec{\zeta }_{ik} (t;\beta _0, B) \mathrm{d} M_{ik}(t) + o_p(1), \end{aligned}$$

where $\varvec{\zeta }_{ik} (t; \beta , B) =\sum _{m=1}^{K} R_{im}(t; \beta ) \phi _{imk}(t; \beta , B) -\varvec{e}_k (t;\beta , B)$. From the arguments in Zhou and Wang (2000), it can be shown that

$$\begin{aligned} \sqrt{n}\widehat{U}_2(\beta _0, B) = -\frac{1}{\sqrt{n}} \sum _{k=1}^{K} \sum _{i=1}^{n} \frac{n-n_k}{n_k} \varvec{F}_{ik}(\beta _0) + o_p(1), \end{aligned}$$

where

$$\begin{aligned} \varvec{F}_{ik}(\beta ) = \int _{0}^{\tau }\varvec{\zeta }_{ik} (t;\beta , B) (r_{ik}(t;\beta )-\psi _{ik}(t;\beta )) Y_{ik}(t)\lambda _{0k}(t) \mathrm{d}t. \end{aligned}$$

Note that $\varvec{F}_{ik}(\beta ) = 0$ if $i \in \overline{V}_k$. Then we have

$$\begin{aligned} \sqrt{n} \widehat{U}(\beta _0, B)&=\frac{1}{\sqrt{n}} \sum _{k=1}^{K} \sum _{i=1}^{n} \int _{0}^{\tau } \varvec{\zeta }_{ik} (t;\beta _0, B) \mathrm{d} M_{ik}(t)\\&\quad -\frac{1}{\sqrt{n}} \sum _{k=1}^{K} \sum _{i=1}^{n} \frac{n-n_k}{n_k} \varvec{F}_{ik}(\beta _0) + o_p(1), \end{aligned}$$

where $M_{ik}(t)$ is a mean-zero martingale and $E(\varvec{F}_{ik}(\beta _0)) = 0$, then by strong law of large numbers, we have $\widehat{U}(\beta _0, B)$ converges to zero with probability 1, thus (ii) is satisfied.

Denote

$$\begin{aligned} \widehat{U}(\beta , B) = \sum _{i=1}^{n} \sum _{k=1}^{K} \widehat{U}_{ik}(\beta , B), \end{aligned}$$

then we can obtain

$$\begin{aligned} \widehat{\varvec{g}}_i(\beta ) = (n \sum _{k=1}^{K} \widehat{U}_{ik}^{\mathrm{T}} (\beta , B_1), n \sum _{k=1}^{K} \widehat{U}_{ik}^{\mathrm{T}} (\beta , B_2), \ldots , n \sum _{k=1}^{K} \widehat{U}_{ik}^{\mathrm{T}}(\beta , B_L))^{\mathrm{T}}, \end{aligned}$$

and

$$\begin{aligned} n \widehat{\varvec{W}}_n(\beta )&= \frac{1}{n} \sum _{i=1}^{n} \widehat{\varvec{g}}_i(\beta ) \widehat{\varvec{g}}_i^{\mathrm{T}}(\beta )\\&= \left( \begin{array}{ccc} \varvec{O}(\beta , B_1, B_1)&{}\cdots &{}\varvec{O}(\beta , B_1, B_L)\\ \vdots &{}\ddots &{}\vdots \\ \varvec{O}(\beta , B_L, B_1)&{}\cdots &{}\varvec{O}(\beta , B_L, B_L) \end{array}\right) , \end{aligned}$$

where

$$\begin{aligned}&\varvec{O}(\beta , B_j, B_{j^{'}})\\&\quad =n\sum \limits _{i=1}^{n} \sum \limits _{k=1}^{K} \sum \limits _{l=1}^{K} \widehat{U}_{ik}(\beta , B_j) \widehat{U}_{il}^{\mathrm{T}}(\beta , B_{j^{'}})\\&\quad =\frac{1}{n} \sum \limits _{i=1}^{n} \sum \limits _{k=1}^{K} \sum \limits _{l=1}^{K} \left\{ \int _{0}^{\tau } \varvec{\zeta }_{ik} (t;\beta , B_j) \mathrm{d} M_{ik}(t)\right\} \left\{ \int _{0}^{\tau } \varvec{\zeta }_{il} (t;\beta , B_{j^{'}})\mathrm{d} M_{il}(t) \right\} ^{\mathrm{T}}+ o_p(1). \end{aligned}$$

Similiarly as in Xue et al. (2010), by condition (C8), we can show that $\varvec{O}(\beta , B_j, B_{j^{'}})$ converges in probability to $\varvec{\omega }(\beta , B_j, B_{j^{'}})$, and $n \widehat{\varvec{W}}_n(\beta )$ converges in probability to $\varvec{W}(\beta ) = [\varvec{\omega }(\beta , B_j, B_{j^{'}})]_{j, j^{'} =1}^{L}$ uniformly for $\beta \in {\mathscr {B}}$, hence (iii) and (iv) are satisfied.

**Asymptotic normality**

Define $\varvec{D}_i(B) = \sum _{k=1}^{K} \int _{0}^{\tau } \varvec{\zeta }_{ik}(t;\beta _0, B) \mathrm{d}M_{ik}(t)$, from the previous statements, for any constant matrix *B*, we can obtain that $\sqrt{n} \widehat{U}(\beta _0, B)$ is asymptotically equivalent to $n^{-1/2} \sum _{i=1}^{n} \varvec{D}_i(B)$, which is a sum of independent *p*-vector random variables with mean zero and variance $\hbox {var}(\varvec{D}_i(B))$. By condition (C8) and the multivariate central limit theorem, we can show that $\sqrt{n} \widehat{U}(\beta _0, B)$ converges in distribution to a normal random vector with mean zero and covariance matrix $\varvec{\omega }(\beta _0, B, B)$, and $\sqrt{n} \widehat{\varvec{G}}_n(\beta _0)$ converges in distribution to a normal random vector, denoted as ${\mathcal {X}}$, with mean zero and covariance matrix $\varvec{W}_0$ as defined in (A.2).

By Taylor expansion of $\widehat{\varvec{Q}}_n^{(1)}(\beta )$ around the true parameter $\beta _0$, we have

$$\begin{aligned} \sqrt{n} (\widehat{\beta }_Q- \beta _0)= - [n^{-1} \widehat{\varvec{Q}}_n^{(2)} (\beta ^{*})]^{-1} [n^{-1/2} \widehat{\varvec{Q}}_n^{(1)}(\beta _0)], \end{aligned}$$

where $\beta ^{*}$ is between $\beta _0$ and $\widehat{\beta }_Q$. From the argument in the proof of Theorem 1 and the consistency of $\widehat{\beta }_Q, n^{-1} \widehat{\varvec{Q}}_n^{(2)}(\beta ^{*})$ and $n^{-1/2} \widehat{\varvec{Q}}_n^{(1)}(\beta _0)$ converge to $2 J_0^{\mathrm{T}} \varvec{W}_0^{-1}J_0$ and $2 J_0^{\mathrm{T}} \varvec{W}_0^{-1} {\mathcal {X}}$ in probability, respectively. Hence, we can obtain that in distribution

$$\begin{aligned} \sqrt{n}(\widehat{\beta }_Q - \beta _0) \rightarrow N(0,(J_0^{\mathrm{T}} \varvec{W}_0^{-1}J_0)^{-1}). \end{aligned}$$

$$\square $$

## References

[CR1] Breslow NE (1972). Discussion of the paper by D. R. Cox. J R Stat Soc Ser B.

[CR2] Cai JW, Prentice RL (1995). Estimating equations for hazard ratio parameters based on correlated failure time data. Biometrika.

[CR3] Cai JW, Prentice RL (1997). Regression estimation using multivariate failure time data and a common baseline hazard function model. Lifetime Data Anal.

[CR4] Cai JW, Shen Y (2000). Permutation tests for comparing marginal survival functions with clustered failure time data. Stat Med.

[CR5] Clayton D, Cuzick J (1985). Multivariate generalizations of the proportional hazard model. J R Stat Soc Ser A.

[CR6] Fan Z, Wang X (2009). Marginal hazards model for multivariate failure time data with auxiliary covariates. J Nonparametr Stat.

[CR7] Greene WF, Cai JW (2004). Measurement error in covariates in the marginal hazards model for multivariate failure time data. Biometrics.

[CR8] Hu C, Lin DY (2004). Semiparametric failure time regression with replicates of mismeasured covariates. J Am Stat Assoc.

[CR9] Liu Y, Zhou H, Cai JW (2009). Estimated pseudopartial-likelihood method for correlated failure time data with auxiliary covariates. Biometrics.

[CR10] Liu Y, Wu Y, Zhou H (2010). Multivariate failure times regression with a continuous auxiliary covariate. J Multivar Anal.

[CR11] Liu Y, Yuan Z, Cai JW, Zhou H (2012). Marginal hazard regression for correlated failure time data with auxiliary covariates. Lifetime Data Anal.

[CR12] Qu A, Lindsay BG, Li B (2000). Improving generalised estimating equations using quadratic inference functions. Biometrika.

[CR13] SOLVD Investigators (1991) Effect of enalapril on survival in patients with reduced left ventricular ejection fractions and congestive heart failure. N Engl J Med 325:293–302 10.1056/NEJM199108013250501 2057034

[CR14] Wei LJ, Lin DY, Weissfeld L (1989). Regression analysis of multivariate incomplete failure time data by modeling marginal distributions. J Am Stat Assoc.

[CR15] Xue L, Wang L, Qu A (2010). Incorporating correlation for multivariate failure time data when cluster size is large. Biometrics.

[CR16] Zhou H, Wang C-Y (2000). Failure time regression with continuous covariates measured with error. J R Stat Soc Ser B.

